# Identification of therapeutic targets applicable to clinical strategies in ovarian cancer

**DOI:** 10.1186/s12885-016-2675-5

**Published:** 2016-08-24

**Authors:** Marianne K. Kim, Natasha Caplen, Sirisha Chakka, Lidia Hernandez, Carrie House, Georgios Pongas, Elizabeth Jordan, Christina M. Annunziata

**Affiliations:** 1 Women’s Malignancies Branch, Center for Cancer Research, National Cancer Institute, Bethesda, MD 20892 USA; 2 Gene Silencing Section, Genetics Branch, Center for Cancer Research, National Cancer Institute, Bethesda, MD 20892 USA; 3 Women’s Malignancies Branch, Center for Cancer Research, National Cancer Institute, 10 Center Drive, Room 4B54, Bethesda, MD 20892-1361 USA

**Keywords:** Ovarian cancer, PLK1, MAP3K7/TAK1, WEE1

## Abstract

**Background:**

shRNA-mediated lethality screening is a useful tool to identify essential targets in cancer biology. Ovarian cancer (OC) is extremely heterogeneous and most cases are advanced stages at diagnosis. OC has a high response rate initially, but becomes resistant to standard chemotherapy. We previously employed high throughput global shRNA sensitization screens to identify NF-kB related pathways. Here, we re-analyzed our previous shRNA screens in an unbiased manner to identify clinically applicable molecular targets.

**Methods:**

We proceeded with siRNA lethality screening using the top 55 genes in an expanded set of 6 OC cell lines. We investigated clinical relevance of candidate targets in The Cancer Genome Atlas OC dataset. To move these findings towards the clinic, we chose four pharmacological inhibitors to recapitulate the top siRNA effects: Oxozeaenol (for MAP3K7/TAK1), BI6727 (PLK1), MK1775 (WEE1), and Lapatinib (ERBB2). Cytotoxic effects were measured by cellular viability assay, as single agents and in 2-way combinations. Co-treatments were evaluated in either sequential or simultaneous exposure to drug for short term and extended periods to simulate different treatment strategies.

**Results:**

Loss-of-function shRNA screens followed by short-term siRNA validation screens identified therapeutic targets in OC cells. Candidate genes were dysregulated in a subset of TCGA OCs although the alterations of these genes showed no statistical significance to overall survival. Pharmacological inhibitors such as Oxozeaenol, BI6727, and MK1775 showed cytotoxic effects in OC cells regardless of cisplatin responsiveness, while all OC cells tested were cytostatic to Lapatinib. Co-treatment with BI6727 and MK1775 at sub-lethal concentrations was equally potent to BI6727 alone at lethal concentrations without cellular re-growth after the drugs were washed off, suggesting the co-inhibition at reduced dosages may be more efficacious than maximal single-agent cytotoxic concentrations.

**Conclusions:**

Loss-of-function screen followed by in vitro target validation using chemical inhibitors identified clinically relevant targets. This approach has the potential to systematically refine therapeutic strategies in OC. These molecular target-driven strategies may provide additional therapeutic options for women whose tumors have become refractory to standard chemotherapy.

**Electronic supplementary material:**

The online version of this article (doi:10.1186/s12885-016-2675-5) contains supplementary material, which is available to authorized users.

## Background

Ovarian cancer is the most aggressive gynecological malignancy among women with more than 20,000 new cases and nearly 15,000 deaths per year in the USA. At diagnosis, most women have advanced disease stage generally due to the lack of signs and symptoms at early stages. The current standard care includes surgical cytoreduction followed by platinum- and taxane-based chemotherapy. Initial cytotoxic chemotherapy effectively achieves complete response in most cases, but relapse within 18 months is common and eventually leading to chemotherapy failure. Therefore, new therapeutic strategies are necessary to improve treatment of recurrent chemotherapy-resistant tumors.

One of the reasons for the high recurrence rate may be the heterogeneity of ovarian cancer. Ovarian cancer can be classified as four major histological subtypes including serous, endometrioid, mucinous, and clear cell and they are believed to be different diseases sharing the same final anatomical location [1]. High grade serous ovarian cancer (HGSOC) is most common and accounts for most deaths in women with ovarian cancers. HGSOC was further classified as mesenchymal, immunoreactive, differentiated, and proliferative based on molecular and genetic profiles [2]. It is characterized by high genomic instability with frequent DNA copy number gains and losses and moderate load of mutations [3]. Advances in understanding molecular aberrations and their pathological signaling have facilitated the use of molecular-driven targeted therapies, and the NCI-MATCH (Molecular Analysis for Therapy Choice) clinical trial has been launched to evaluate the effectiveness of cancer treatment according to molecular abnormalities.

Loss-of function screens by shRNA/siRNA provide a useful tool to identify novel therapeutic targets in the laboratory. For example, a recent synthetic lethality screen suggested a mechanistic explanation of mutual exclusivity between CCNE1 amplification and BRCA1/2 mutation, and further showed the sensitivity of CCNE1-amplified tumor cells to bortezomib [4]. Another in vivo shRNA screen identified BRD4 as a therapeutic target, demonstrating that BRD4 inhibitor (JQ1) decreased survival of high MYC-expressing ovarian cancer cells [5]. We previously performed two independent shRNA screens to investigate the functional role of NF-kB signaling in ovarian cancer cell proliferation and survival [6, 7]. In one of these screens, CHEK1 loss sensitized ovarian cancer cells to IKKε loss [6]. We proceeded to evaluate the combined efficacy of CHEK1 inhibitor with topotecan, a salvage treatment for platinum-resistant ovarian cancer, showing a synergistic cytotoxic effect with reduced dosages of both drugs [8]. These results suggest that molecular-based therapy may improve the efficacy of currently available treatments, while possibly reducing side effects by lowering the effective concentration required to achieve tumor response.

In the current study, we re-focused our efforts to identify targets essential for OC survival, independent of NF-kB. Herein we show our prioritization strategy from shRNA screens, further evaluation by siRNA knock-down and chemical inhibitors, and recommended combination schema in an expanded set of ovarian cancer cell lines.

## Methods

### Chemical inhibitors

Stock solutions of 50 mM Lapatinib (GW572016) Ditosylate (Selleck, S10128), 10 mM MK1775 (Selleck, S1525), 10 mM BI6727 (Selleck, S2235), and 10 mM Oxozeaenol (Tocris, cat. no 3604) were prepared in DMSO except 5 mM Cisplatin (Tocris, cat no 2251) in PBS, and aliquots were stored at −80 °C. The highest final concentration of DMSO in the culture in this study was 0.1 % which caused no cellular toxicity in ovarian cancer cells. All working stocks were diluted in complete medium.

### Cell lines

All ovarian cancer cell lines in this study were previously described including the source and authentication of the cell lines, and maintained in RPMI supplemented with 10 % heat-inactivated FBS [9].

### The cancer genome atlas data

TCGA ovarian cancer dataset was analyzed and extracted using a web-based tool (http://www.cbioportal.org/public-portal/).

### Validation screen by siRNAs

All siRNAs were purchased from Qiagen, and their IDs, sequences and plate layout were shown in Additional file 1: Table S1. For each cell line, seeding cell number and lipid volume were determined for optimal transfection efficiency. Cells were seeded at 750 cells/well except Skov3 at 500 cells/well, and transfected with 0.06 μl RNAiMax except Skov3 with 0.07 μl. siRNA transfection was performed at a final concentration of 20 nM, and 2 μl of 400 nM stock siRNAs were spotted on 384 well plates. These spotted siRNA plates were stored at −80 °C until used. Twenty μl of serum free RPMI containing RNAiMax was added and plates were incubated at room temperature for 15 min followed by adding cells re-suspended in 20 μl of RPMI containing 20 % FBS. The plates were then incubated at room temperature for 30 min before putting at 37 °C. Ovcar8, Ovcar3, Skov3, and Igrov1 cells for 3 days, A2780 for 2 days, and Ovcar5 cells were incubated for 4 days allowing approximately two doubling times for optimal cellular viability assays. The screening in each cell line was independently repeated in three plates. AllStars Neg. control siRNA from Qiagen (cat # 1027281) was used as a negative control (siNeg) and AllStars Hs Cell Death Control siRNA (cat # 1027299) was used as a positive control. Cellular viability was determined using CellTiter Glo (20 μl/well) and the average viability from three plates was normalized by the average of siNeg from each plate.

### Viability assay

Cells were seeded in 96-well plates at a density of 1–2000 cells/50 μl/well in triplicates. In general, the drug in 50 μl was added 24 h after seeding and XTT assay was routinely performed in 3 days after drug treatment unless indicated. Cellular viability was assessed by incubating cultures with 25 μl of XTT freshly mixed with PMS (Sigma) and absorbances were read in a Tecan plate reader (Research Triangle Park, NC). Cellular proliferation was calculated relative to experimental negative controls and standard deviation was calculated from triplicates. Based on XTT numbers, IC50s were calculated using CompuSyn software [10]. Briefly, the median effect dose (Dm) is obtained from the anti-log of the x-intercept of the median effect plot: log(Fa/Fu) = m*log(D) - m*log(Dm) where Fa is Fraction affected, Fu is Fraction unaffected, m is slope [11]. Viability assay in SCM (stem cell media) was done using CellTiter Glo Luminescent Cell Viability Assay according to manufacturer’s instructions (Promega).

### Western blot analysis

Total protein was extracted from sub-confluent cells with 1 % NP40 lysis buffer containing 150 mM NaCl, 50 mM TrisHCl, 10 % glycerol, 1 X Halt proteinase inhibitor cocktail, 5 mM NaF, and 1 mM NaOrthovanadate. Protein concentrations were estimated using BCA Protein Assay Kit (Thermo Scientific, Rockford, IL). The proteins were separated on the NuPage 4–12 % gel (Invitrogen, Carlsbad, CA) and the band was visualized using either Luminata Classico or Crescendo Western HRP substrate system (Millipore) depending on the signal intensities. Antibodies c-ErbB2/c-Neu (Calbiochem, cat. no. OP15L), WEE1 (Santa Cruz, sc-5285), PLK1 (Millipore, #05-844), TAK1 (Santa Cruz, sc-166562), and GAPDH (Millipore, MAB374) were used, and the secondary antibodies ECL anti-rabbit IgG HRP and ECL anti-mouse IgG HRP (GE Healthcare) were used at 1:5000 dilutions.

### Flow cytometry analysis

Ovcar5 and Ovcar8 cells were grown for 1 week in the presence of traditional RPMI culture media containing 10 % FBS or in serum-free stem cell media containing 20 ng/ml EGF and 10 ng/ml FGF. Cultures were maintained for 7 days (with media change at day 3) before performing flow cytometry. Approximately 5 × 10^5^ cells were analyzed for ALDH activity and CD133 expression. ALDH activity was evaluated using the Aldefluor Kit (StemCell Technologies) according to manufacturer’s instructions. Following staining procedure for ALDH, cells were incubated with APC conjugated CD133 antibody (Miltenyi Biotec) 1:11 in the Aldefluor assay buffer for 30 min on ice protected from light.

### Statistical analysis

Statistical analysis for Fig. 2a was performed using the Mann-Whitney *U* test in IBM SPSS Statistics Version 21. Cellular viability for each siRNA construct in each of the six cell lines was compared to that with siNeg in triplicate experiments; *p* < 0.05 was considered statistically significant. Error bars represent the standard error of the mean. For Fig. 2b, shRNA counts for a given shRNA in a given experiment were normalized within an experiment by pool and then fitted with a negative binomial extension of the Poisson distribution, with parameters fitted iteratively with the mean estimate via maximum likelihood and the method of moments used to estimate the dispersion [12]. *P*-values for differences between experiments were found by comparing the log of the estimated normalized averages between experiments with a normal approximation to the error. In comparing an experiment where a given shRNA had 0 counts across all replicates resulting in an infinite estimate of the log average, the *p*-value reported was the likelihood of getting 0 counts across all replicates given the negative binomial model of the shRNA in the experiment to which it was being compared. Statistical analysis for Fig. 5a–e was performed using the One-Way ANOVA test with a Tukey post-hoc test in IBM Statistics Version 21. Relative cellular viability of each experimental condition was compared to control; *p* < 0.05 was considered statistically significant. Error bars represent the standard deviation of the mean.

## Results

### Re-analysis of shRNA-mediated lethality screens identified 55 kinases essential for OC survival

**Fig. 1:**
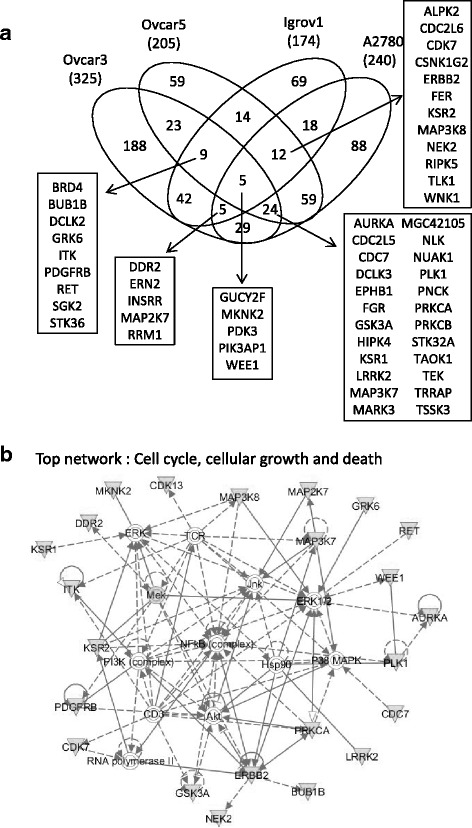
Re-analysis of shRNA screens in an unbiased manner **a** Target genes from shRNA screens in four cell lines are compared and common targets are shown in venn diagram. **b** Fifty five genes are uploaded onto Ingenuity Pathway Analysis and the most significant network is shown

Our previous lethality screenings were performed in Ovcar5 and A2780 using shRNA library directed against the human kinome [6], and in Ovcar3 and Igrov1 using whole genome shRNA library [7] in the context of NF-kB signaling. In the current study, we reanalyzed these data from four different cell lines in an unbiased manner to identify genes required for OC survival. In each cell line, comparisons were made at two different time points relative to Day 0, and candidate shRNAs were selected if identified in both comparisons with a *p* value of less than 0.05 and a fold change of less than 0.7. With this cut-off, 305 (240 genes), 253 (205 genes), 190 (174 genes), and 404 (325 genes) shRNAs were selected in A2780 (Additional file 2: Table S3), Ovcar5 (Additional file 3: Table S4), Igrov1 (Additional file 4: Table S5), and Ovcar3 (Additional file 5: Table S6), respectively. Under the most stringent cut-off of four out of four cell lines, five genes including *GUCY2F*, *MKNK2*, *PDK3*, *PIK3AP1*, and *WEE1* were identified as essential for OC cell survival (Fig. 1a). When the stringency of analysis was relaxed to allow three out of four cell lines affected, a total of 55 genes were included. The most significant cellular functions regulated by these 55 genes were cell cycle, and cancer cell death and survival, as determined by Ingenuity Pathway Analysis (Fig. 1b). Next, we examined the expression levels of these 55 genes in OCs in The Cancer Genome Atlas (TCGA) to estimate clinical relevance associated with their expression [2]. Fourteen genes were overexpressed with a cut-off of log2 ratio greater than 0.5 in more than 40 % of the tumors compared to nine normal controls, while 11 genes were interestingly underexpressed with a cut-off of log2 ratio less than −0.5 in more than 40 % of tumors (Table 1). Although it seems unreasonable to see that overexpression and underexpression of a gene would produce similar phenotype, this might be due to the perturbation of physiological balance by either overexpression or underexpression of each gene, contributing to tumorigenesis. Of note, TCGA dataset consists of ovarian serous adenocarcinoma, and our candidate genes were identified from two serous (Ovcar5 and Ovcar3) and two non-serous (A2780 and Igrov1) cell lines. With this in mind, we further validated the functional significance of these 55 genes by a short-term knockdown using siRNA.

**Table 1:** The percentage of ovarian cancer tumors in TCGA are shown with a cut-off of log2 tumor/normal ratio in more than 40 % tumors detected by Agilent G4502A microarray chips

Gene	Log2 > 0.5	Gene	Log2 < −0.5
ALPK2	54 %	CSNK1G2	63 %
AURKA	97 %	DDR2	80 %
BUB1B	96 %	GUCY2F	97 %
CDC7	89 %	ITK	50 %
EPHB1	67 %	LRRK2	92 %
GRK6	44 %	MGC42105	46 %
KSR2	41 %	MKNK2	46 %
MAP3K7	45 %	PDGFRB	49 %
NEK2	97 %	PRKCB	61 %
NLK	59 %	RET	41 %
PIK3AP1	58 %	TEK	91 %
PLK1	58 %		
RRM1	41 %		
TLK1	42 %		

The data shown are from the dataset of Nature publication [2]. The data were extracted from TCGA analysis with RMA normalized log2 ratio of tumor to nine normal controls

### siRNA-mediated validation identified pro-survival essential targets in OC cancer lines

**Fig. 2:**
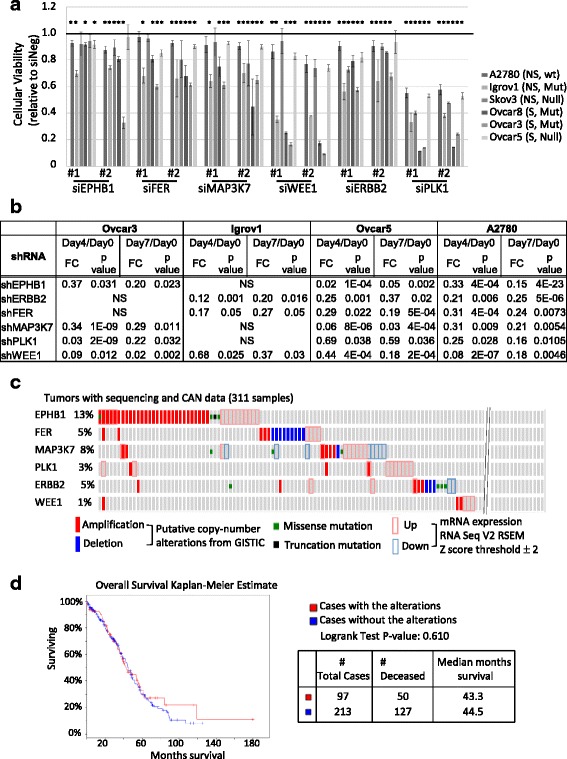
Validation of shRNA candidate genes by siRNAs in six ovarian cancer cell lines **a** siRNA screens are done in three serous (S) and three non-serous (NS) cell lines. p53 status is indicated; Ovcar3 (Mut: P72R, R248Q), Ovcar8 (Mut: amino acid deletion: aa126-132), Igrov1 (Mut: Y126C), Ovcar5 and Skov3 (Null, no p53 detected on Western blot), A2780 (wide type) [6]. The value of each siRNA from CellTiter Glo assay was normalized by the average value of siNeg in each plate to calculate relative cellular viability, and then the average of three normalized values were plotted with standard deviation. The red bar is drawn at 1.0 which means no toxicity upon knockdown. Cellular viability for each siRNA construct across the six cell lines was compared to that with siNeg; * indicates *p* < 0.05 (Mann Whitney *U*-test). Error bars represent the standard error of the mean for each cell line, per siRNA. **b** Target shRNA depletion (shown as fold change: FC) at two different time points compared to day 0 (baseline control) are shown. shRNA screens were done in four biological replicates in Ovcar5 and A2780, and in six biological replicates in Ovcar3 and Igrov1 [6, 7]. *p*-value compares the log of the estimated normalized averages between experiments (see methods). **c** Genetic alterations of six candidate targets were examined in TCGA ovarian tumor samples with sequencing and CNA data using a web-based cBioPortal tool (http://www.cbioportal.org/public-portal/). **d** Overall survival analysis based on the alterations of six candidate targets in OC was extracted from TCGA data analysis

We hypothesized that if the targets could be validated by different methods and in multiple cell lines, in spite of the extremely heterogeneous genetic backgrounds, it would be stronger and have more widely applicable values in ovarian cancer. In order to validate and prioritize candidate genes, we compared the effect of target suppression by shRNA (long term stable loss), siRNA (short term acute and transient loss), and then chemical inhibitors. We employed siRNA lethality assay in an expanded set of 6 OC cell lines additionally including Ovcar8 (serous) and Skov3 (non-serous). Two siRNAs per gene were tested in a 384-well format as outlined (Additional file 1: Table S1). Transient transfection protocols such as seeding cell numbers and lipid to siRNA ratio were optimized in each cell line using positive (AllStars Hs Cell Death Control) and negative (AllStars siNeg. control) siRNA controls (Additional file 6: Figure S1). siRNA screen in each cell line was done in three independent plates rather than three replicates in one plate. This resulted in bigger standard deviations in general, but this design would minimize false positives. We selected targets with below 0.95 of the average decreased viabilities of two siRNAs compared to siNeg control from the three independent replicates in all 6 OC cell lines (Additional file 7: Table S7). Based on these criteria, six genes (*EPHB1*, *FER*, *MAP3K7*, *PLK1*, *ERBB2*, and *WEE1*) were identified in all six cell lines (Fig. 2a). Cellular viability for the selected siRNA constructs in each of the six cell lines was statistically significantly decreased as compared to siNeg (Additional file 7: Table S7). Of note, analysis of both constructs on individual cell lines reached statistical significance in the majority of cases. Since the 55 candidate targets were selected based on a three out of four cell line criteria in shRNA screens, it was not surprising to observe no significant lethal effects in some cell lines in these siRNA screens. The degree of cytotoxicity of knockdown was greater by shRNA than by siRNA in general, possibly due to stable selection of shRNA, although this was not always true for every gene in each cell line such as shPLK1 in Igrov1 (Fig. 2b). Regardless of OC subtypes (serous vs. non-serous) or the status of p53, the loss of PLK1 or WEE1 was generally more detrimental than that of the other genes. In TCGA dataset, the number of individuals with alterations in each gene was small, and their alterations had no statistically significant association with overall survival (Fig. 2c, d). Additionally, overall survival analysis of each gene alone did not produce a statistically significant association either.

### Pharmacological inhibitors generally recapitulated siRNA lethality

**Fig. 3:**
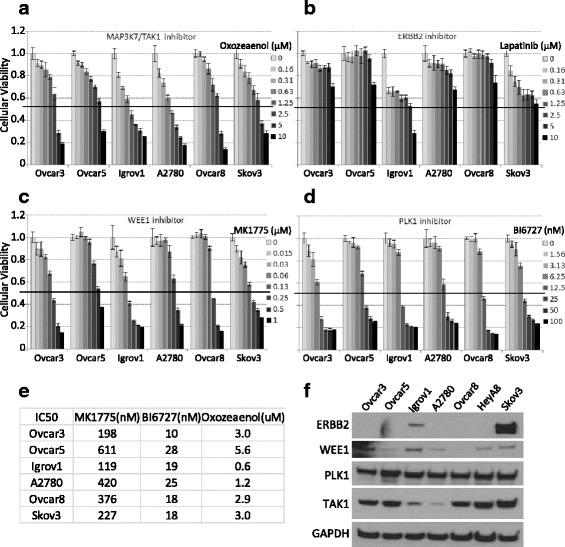
Evaluation of cellular toxicities upon pharmacological intervention **a**–**d** Cells were seeded at 1000 cells/well in 50 μl, 20–24 h prior to the addition of the drug in 50 μl. XTT assay was performed 3 days later upon drug treatment. The viability was calculated relative to no drug treatment and the error bars represent standard deviations calculated from three replicates. **e** IC50 values were calculated by CompuSyn after converting relative viability values to fraction affected numbers. **f** The steady-state level of each inhibitor target protein was examined by Western blotting analysis. Total 40 μg of proteins was separated on 4–12 % gradient gel and GAPDH was used as a loading control

To move these findings towards the clinic, we investigated whether pharmacological inhibitors could resemble the lethal effect of the loss-of function by siRNA. We chose four inhibitors: oxozeaenol (for MAP3K7/TAK1), lapatinib (ERBB2), MK1775 (WEE1), and BI6727 (PLK1) based on the availability of pharmacological compounds and clinical applicability. We measured cell sensitivity to inhibitor in a 3-day assay (Fig. 3a–d). The ranges of IC50s were 0.6–6 μM, 120–610 nM, and 10–35 nM for oxozeaenol, MK1775, and BI6727, respectively (Fig. 3e, Additional file 8: Figure S2). Interestingly, all cell lines were cytostatic to lapatinib, including *ERBB2*-amplified Skov3, suggesting that a kinase independent function of ERBB2 may play a role in OC cell survival. Since lapatinib also inhibits EGFR, we double-checked the shRNA data to see the effect of stable knockdown of EGFR in all four cell lines (Ovcar3, Igrov1, Ovcar5, and A2780) and found that shEGFR did not affect the cellular viability with the statistical cut-off used in this study. This suggests that EGFR may not facilitate cellular viability, at least in these cell lines. The steady-state level of each target protein varied across cell lines (Fig. 3f). Interestingly, ERBB2 expression was detected in only Igrov1 and Skov3. OC cells showed different levels of WEE1 expression presenting no clear correlation with the sensitivity to MK1775, while they showed abundant expressions of TAK1 and PLK1 and were sensitive to oxozeaenol and BI6727. Taken together, inhibitors of PLK1, TAK1, and WEE1 were potent in killing OC cells, consistent with the siRNA findings. In addition, OC subtypes (serous or non-serous) or the steady-state levels of target proteins could not predict the sensitivities to these inhibitors.

### Cisplatin-resistant cells or stem-like population are sensitive to PLK1 inhibition

**Fig. 4:**
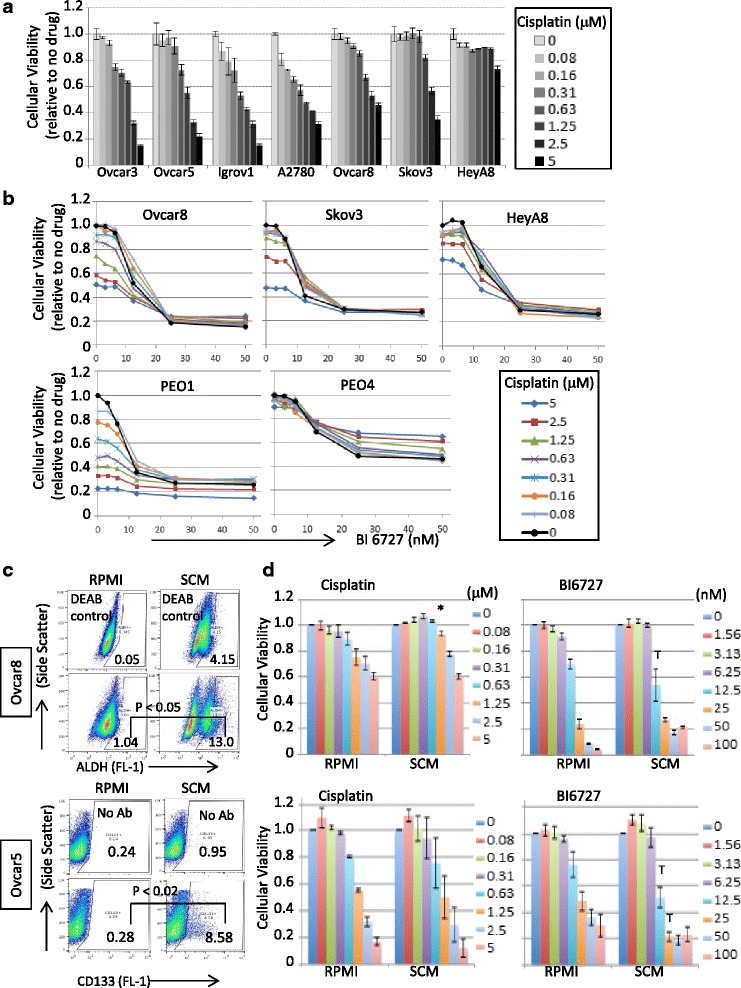
Cytotoxic effect of BI6727 in the presence of cisplatin **a** Cells were seeded at 1000 cells/well in 50 μl, 20–24 h prior to the addition of the drug in 50 μl. XTT assay was performed 3 days later upon drug treatment. The viability was calculated relative to no drug treatment and the error bars represent standard error calculated from three replicates. **b** XTT assays were performed as described in Fig. 4a except using two inhibitors of Cisplatin and BI6727 in 50 μl. PEO1 and PEO4 were seeded at 2000 cells per well. **c** CD133 surface expression and ALDH1 activity were measured by flow cytometry in Ovcar5 and Ovcar8 cells grown in either RPMI or SCM. The data are compiled from 4 (for Ovcar5) and 3 (for Ovcar8) independent experiments. For statistical analysis, the differences in positive population of markers were calculated by 2-tailed *t*-test **d** Ovcar5 and Ovcar8 cells (2 × 10^3^ cells/well) were seeded on white plates in RPMI media containing 10 % FBS or in serum-free stem cell media containing 20 ng/ml EGF and 10 ng/ml FGF. Twenty-four hours after seeding, cells were treated with cisplatin or BI6727. The viability was measured using the CellTiter Glo and calculated relative to no drug treatment and the error bars represent standard error calculated from three experiments. * *p* < 0.05 by one-way ANOVA with Tukey post-hoc test. T (trend), *p* ≤ 0.2

OC initially responds to platinum chemotherapy treatment, but most cancers eventually relapse and become resistant to standard agents including cisplatin. In the current study, we found that most cell lines highly express PLK1 protein, and the PLK1 inhibitor BI6727 potently killed OC cell lines (Fig. 3d and f). Therefore, we tested whether BI6727 would sensitize cisplatin resistant OC cells. We first examined the cytotoxicity of cisplatin in a panel of 7 OC cell lines, and observed that Ovcar8, Skov3, and HeyA8 were relatively resistant to cisplatin with IC50s of greater than 1 μM (Fig. 4a, Additional file 9: Figure S3). When combined with BI6727, cisplatin did not enhance the cytotoxicity of BI6727 except at high concentrations if any (Fig. 4b). PEO1 and PEO4 are a pair of high grade serous OC cell lines established from the same patient before and after platinum-based chemotherapy [13]. We found that PEO1 was very sensitive to single treatment of either drug alone, while PEO4 was resistant to cisplatin. Of note, cisplatin attenuated the cytotoxicity of BI6727 in PEO4 (Fig. 4b). These data suggest that combining BI6727 with cisplatin to treat cisplatin resistant OC patients may not be clinically beneficial.

Cisplatin-resistant cells have the ability to re-populate tumors and cause disease recurrence. We hypothesized that PLK1 inhibitor BI6727 could reduce the tumor-initiating cell population due to its ability to kill cisplatin-resistant cells. We attempted to enrich for this tumor-initiating or cisplatin resistant cell population by culturing Ovcar5 and Ovcar8 cell lines in stem cell medium (SCM) [14, 15]. CD133 expression and/or ALDH1 activity are potential markers of the stem-like population [15–18]. OC cell lines in SCM showed statistically significant increases in CD133 (Ovcar5) or ALDH (Ovcar8) positive population from three to four independent experiments (Fig. 4c). As expected, Ovcar8 cells were slightly more resistant to cisplatin when cultured in SCM, while both cell lines showed similar sensitivity to BI6727 in both conditions, with a trend towards increased sensitivity in the SCM condition (Fig. 4d, Additional file 10: Figure S4). This suggests that BI6727 is potent to kill cells independent of cisplatin responsiveness and may be effective in stem-like population.

### Combined inhibition at low concentrations killed OC cells more effectively than single treatment at high concentrations

**Fig. 5:**
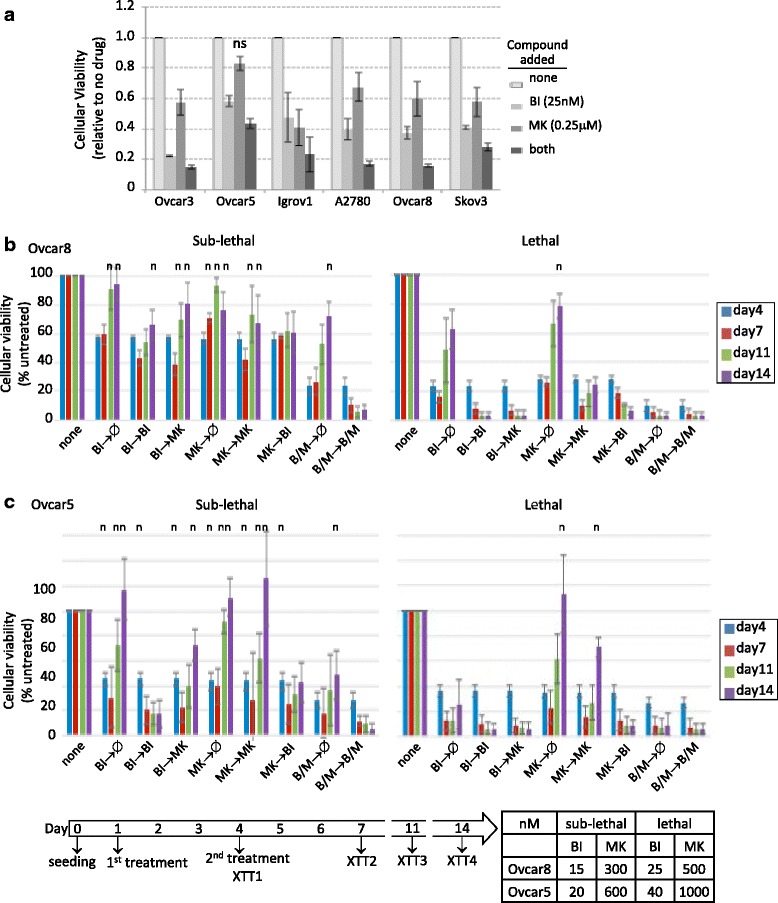
Cytotoxicity of different combination treatments **a** Cells were seeded at 2000 cells/well in 50 μl, 20–24 h prior to the addition of the drug in 50 μl. XTT assay was performed 3 days later upon drug treatments of PLK1 and TAK1 inhibitors. Statistical comparisons were calculated using two-way ANOVA with Dunnett post-hoc test, and *p* < 0.05 considered significant. All experimental conditions resulted in statistically significant differences from no-treatment control, except for MK alone in OVCAR5, indicated by ns. **b**, **c** OVCAR8 cells (**b**) or OVCAR5 cells (**c**) were seeded at 1000 cells/well in 50 μl. First drug was added in a 50 μl volume and then second drug was added in a 80 μl volume producing the indicated final drug concentration after removing 80 μl from each well. For days 7 and 14 time point plates, 150 μl of fresh complete medium was added after taking 150 μl of old culture. At Day11, fresh medium was added to day 14 plates in the same way. Of note, the cellular viabilities of untreated cells at days 11 and 14 were saturated and might not be accurately reflect in these graphs. The charts display compiled data from three independent experiments. For **b** and **c**, n indicates non-significant difference (*p* > 0.05) compared to no treatment, based on the Two-Way ANOVA test with a Dunnett post-hoc test

Single agent therapies often result in resistance and relapse, and combination treatments may have a higher chance of success with a better therapeutic index. WEE1 and PLK1 are involved in the G2/M phase of cell cycle regulation, and TAK1 is an upstream activator of the tumor-promoting NF-kB signaling. We hypothesized that combined kinase inhibition, either within one pathway or targeting independent pathways, may provide more potent cellular cytotoxicity than single agents alone. We proceeded to test the cytotoxic effect of WEE1 and PLK1 inhibitors, by measuring cellular viabilities after exposing cells to low concentrations of the drug pair in six cell lines (Fig. 5a, Additional file 11: Figure S5A). Concentrations were chosen to be below the IC50 of each compound when administered as a single agent (see Fig. 3). In general, the combination of inhibitors killed more cells than single drug in all six cell lines, but the effect was somewhat dependent on cell line. Cellular viability with single drug treatment was compared to either no treatment or dual treatment, and was found to be statistically significantly different (*p* < 0.05, two-way ANOVA test with a Dunnett post-hoc test). Importantly, the combination effectively killed cisplatin resistant cell lines such as Ovcar8 and Skov3.

Since BI6727 and MK1775 are currently under clinical development, we further focused on these PLK1 and WEE1 inhibitors to examine the benefit of their combined treatment over an extended time period. We treated cells at sub-lethal (IC50) and lethal (IC80) concentrations either once or twice in different orders as indicated (Fig. 5b, c). At day 7, the culture medium was removed and replaced with fresh complete medium. Cellular re-growth upon drug withdrawal was measured at days 11 and 14. Exposing cells to a single dose of the co-treatment or two doses of single treatment at sub-lethal concentrations allowed subsequent re-growth of cancer cells when the drugs were no longer present (Fig. 5b, c, left panel, Additional file 11: Figure S5B, 5D). Interestingly, the cells treated with MK1775 were recovered more quickly than those with BI6727. At lethal single-agent concentrations, single dose of either drug still showed cellular re-growth, but twice treatments with BI6727 or BI6727/MK1775 in either order prevented re-growth (Fig. 5b, c, right panel, Additional file 11: Figure S5C, 5E). Interestingly, two doses of MK1775 treatment initially achieved maximal cytotoxicity, but the cellular viability recovered after the drug was washed off. Most importantly, twice co-treatments with BI6727 and MK1775 at sub-lethal concentrations achieved maximal cytotoxic activities and the cells did not grow back after the drugs were washed off. Taken together, this result provides a strong rationale to combine these two drugs although the combination was not synergistic in all ovarian cancer cell lines tested. In summary, these data suggest that the combined treatment with BI6727 and MK1775 at sub-lethal concentrations may be efficacious to treat ovarian cancer, achieving reduced toxicities and avoiding recurrence.

## Discussion

By re-analyzing our previous shRNA screens, we identified 55 genes required for ovarian cancer survival. Not surprisingly, these genes are known to regulate cell cycle, cell death and survival. Their pro-survival functions were validated by siRNA-mediated depletion in a panel of ovarian cancer cell lines representing different histologies. While the shRNA screens were performed over 1–2 weeks with stable knockdown, the siRNA validation screens were carried out by transient transfection over about two cellular doubling times. Therefore, the validated targets by siRNA depletion are likely to be involved in direct and immediate cellular survival role. Among those genes, we focused on ERBB2, TAK1, WEE1, and PLK1 based on clinical application and availability of pharmacological inhibitors. Consistent with our findings, PLK1 was also identified in a recent in vivo shRNA screen in ovarian cancer [5].

Despite successful OC cell killing with siRNA knockdown of ERBB2, the ERBB2 inhibitor lapatinib showed very limited cellular toxicity. It is unclear why the gene knockdown was so effective, when protein expression was negligible in most cell lines. We used the monoclonal antibody produced against a c-terminal synthetic peptide (Calbiochem, OP15L) to measure the protein, leaving the possibility that even cells without detectable expression may express different isoforms on which they depend for survival. It is also possible that ERBB2 isoforms function in a kinase independent manner in ovarian cancer cells. In either case, selection of ERBB2 inhibitor for treatment of ovarian cancer should not be based solely on its level of gene expression. Also, a recent clinical study agrees with our finding, showing that lapatinib had a minimal activity and only a small fraction of ovarian cancer overexpressed EGFR and ERBB2 (HER2) [19].

Consistent with our siRNA data, the MAP3K7/TAK1 inhibitor oxozeaenol was toxic to OC cell lines in the range of 1–5 μM. TAK1 can act as an upstream regulator of the NF-kB signaling promoting ovarian cancer growth and metastasis [20]. Although this chemical inhibitor is a selective and potent inhibitor of TAK1, its use is limited to preclinical in vitro and in vivo models. On the other hand, the WEE1 inhibitor MK1775 has been actively tested in leukemia and many solid tumors including ovarian cancer as mono- or combined therapy (clinicaltrials.gov). For example, MK1775 is currently under evaluation in combination with either gemcitabine or paclitaxel and carboplatin to treat refractory or resistant ovarian cancer or platinum-sensitive p53 mutated ovarian cancer.

PLK1 inhibitor, BI6727 (volasertib) is currently registered in 20 different clinical trials, and one study in ovarian cancer has been completed (NCT01121406). In this trial, none of patients in the volasertib arm completed the treatment course due to progressive disease or adverse effects. Another clinical trial (NCT00969761) using BI6727 in combination with either cisplatin or carboplatin in advanced and metastatic solid tumors is completed, but the study reports are not yet available. Based on our preclinical in vitro data, combining BI6727 with cisplatin did not result in additional cytotoxicity, and the combination was even possibly antagonistic in platinum-resistant PEO4 cells. These results provide a potential explanation as to why the prior clinical trial was unsuccessful. On the other hand, the combination of BI6727 with WEE1 inhibitor MK1775 resulted in cytotoxic activity at concentrations lower than those required to kill cells as single agents. Furthermore, the combined effect was maintained even after the drugs were washed off. These findings support moving forward with combined WEE1/PLK1 inhibition as a promising new clinical strategy for the treatment of women with platinum-refractory ovarian cancer.

Clinical benefit for women with relapsed platinum refractory or resistant ovarian cancer is typically defined as objective response or disease stabilization for greater than 6 months. These criteria are typical endpoints in phase two trials testing potential new therapies for women recurrent ovarian cancer [21]. Numerous drugs have been tested in the setting of platinum-resistant ovarian cancer but unfortunately response rates achieved were less than 6–20 % with short duration of responses (12–17 weeks). While it is difficult to extrapolate from in vitro tumor cell line suppression to long term clinical benefit in patients, the lack of tumor cell re-growth after combined PLK1 and WEE1 inhibition in our study suggest that this could be an interesting strategy to develop further. Another consideration for clinical development is the occurrence of side effects in patients. Again, while it is difficult to predict clinical toxicity based on in vitro studies, it is likely that lower doses of drugs would minimize side effects. In our study, the sequential exposure of low-dose combined drugs achieved similar tumor cell control as higher doses. This suggests an effective and tolerable treatment regimen to develop for women with relapsed ovarian cancer.

## Conclusions

Loss-of-function screens followed by in vitro target validation using chemical inhibitors identified clinically relevant targets for ovarian cancer. This approach has the potential to systematically refine therapeutic strategies for treating a deadly disease. Molecular target-driven strategies may provide additional therapeutic options for women whose tumors have become refractory to standard chemotherapy.

## Additional files

Additional file 1: Table S1. Qiagen siRNA IDs, their sequences, and plate layout. (XLS 53 kb) Additional file 2: Table S3. Candidate shRNAs identified in Ovcar5. (XLS 426 kb) Additional file 3: Table S4. Candidate shRNAs identified in Igrov1. (XLS 262 kb) Additional file 4: Table S5. Candidate shRNAs identified in Ovcar3. (XLS 55 kb) Additional file 5: Table S6. siRNA validation screen result of 55 candidate targets in 6 ovarian cancer cell lines. (XLS 90 kb) Additional file 6: Figure S1. Transfection optimization for siRNA screen. (PPT 235 kb) Additional file 7: Table S7. Mann Whitney *U*-test for significance related to Fig. 2a. (DOCX 15 kb) Additional file 8: Figure S2. Dose-effect curves and output from median effect plot for MK1775, BI6727 and Oxozeaenol. (PPT 259 kb) Additional file 9: Figure S3. Two-way ANOVA with REGWQ post-hoc test for cisplatin dose effect. (PPTX 65 kb) Additional file 10: Figure S4. Two-way ANOVA with Tukey post-hoc comparing effect of culture conditions and cisplatin or BI6727. (PPTX 112 kb) Additional file 11: Figure S5. Two-way ANOVA with Dunnett post-hoc comparing sequential exposure to BI6727 and MK1775. (PPTX 70 kb) Additional file 12: Table S2. Candidate shRNAs identified in A2780. (DOCX 40 kb)
