# Supplementary material for: Identification of therapeutic targets applicable to clinical strategies in ovarian cancer
Source: BMC Cancer. 2016 Aug 24;16(1):678. doi: 10.1186/s12885-016-2675-5 (PMC4997769; doi:10.1186/s12885-016-2675-5)
Supplement: Additional file 6: Figure S1. — Transfection optimization for siRNA screen. (PPT 235 kb) [file 12885_2016_2675_MOESM6_ESM.ppt]

## Slide 1
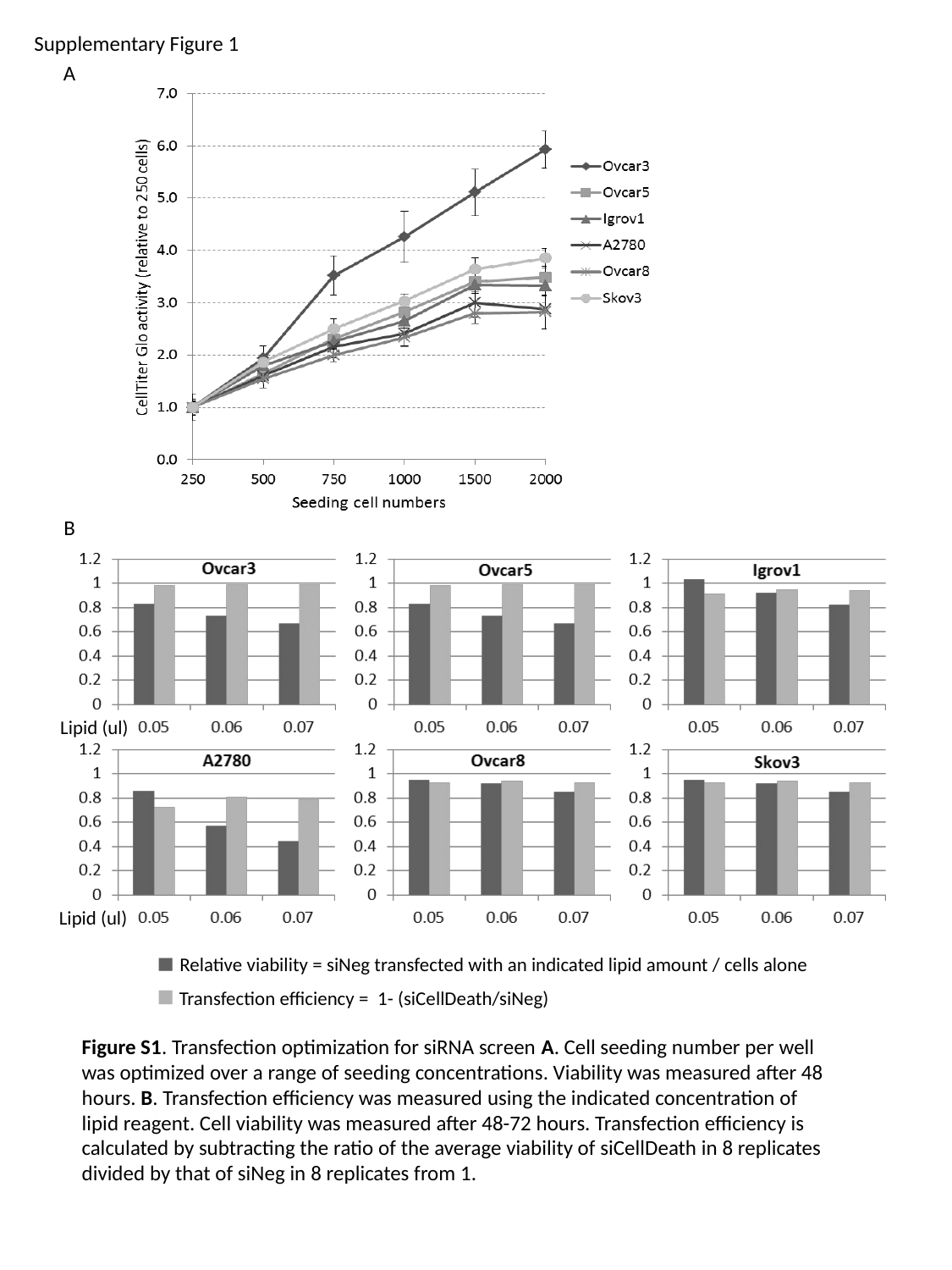

Supplementary Figure 1
A
B
Lipid (ul)
Lipid (ul)
Relative viability = siNeg transfected with an indicated lipid amount / cells alone
Transfection efficiency = 1- (siCellDeath/siNeg)
Figure S1. Transfection optimization for siRNA screen A. Cell seeding number per well was optimized over a range of seeding concentrations. Viability was measured after 48 hours. B. Transfection efficiency was measured using the indicated concentration of lipid reagent. Cell viability was measured after 48-72 hours. Transfection efficiency is calculated by subtracting the ratio of the average viability of siCellDeath in 8 replicates divided by that of siNeg in 8 replicates from 1.
