# Supplementary material for: Identification of therapeutic targets applicable to clinical strategies in ovarian cancer
Source: BMC Cancer. 2016 Aug 24;16(1):678. doi: 10.1186/s12885-016-2675-5 (PMC4997769; doi:10.1186/s12885-016-2675-5)
Supplement: Additional file 7: Table S7. — Mann Whitney U-test for significance related to Fig. 2a. (DOCX 15 kb) [file 12885_2016_2675_MOESM7_ESM.docx]

**Additional file 7: Table S7:** Statistical analysis for cellular viability: Mann-Whitney U-test was performed for cellular viability for each cell line and each siRNA compared against siNeg. Listed are the asymptotic significance (2-tailed) p-values for each comparison.

| **siRNA** | **siEPHB1** | | **siFER** | | **siMAP3K7** | | **WEE1** | | **ERBB2** | | **PLK1** | |
| --- | --- | --- | --- | --- | --- | --- | --- | --- | --- | --- | --- | --- |
|  | **#1** | **#2** | **#1** | **#2** | **#1** | **#2** | **#1** | **#2** | **#1** | **#2** | **#1** | **#2** |
| A2780 | 0.023 | 0.008 | 0.27 | 0.021 | 0.078 | 0.017 | 0.014 | 0.005 | 0.014 | 0.021 | 0.004 | 0.004 |
| IGROV1 | 0.004 | 0.004 | 0.004 | 0.009 | 0.004 | 0.004 | 0.004 | 0.004 | 0.004 | 0.004 | 0.004 | 0.004 |
| SKOV3 | 0.558 | 0.045 | 0.255 | 0.025 | 0.972 | 0.025 | 0.704 | 0.004 | 0.004 | 0.012 | 0.004 | 0.004 |
| OVCAR8 | 0.016 | 0.004 | 0.004 | 0.004 | 0.004 | 0.004 | 0.004 | 0.004 | 0.004 | 0.004 | 0.004 | 0.004 |
| CAOV3 | 0.27 | 0.004 | 0.004 | 0.004 | 0.004 | 0.004 | 0.004 | 0.004 | 0.004 | 0.004 | 0.004 | 0.004 |
| OVCAR5 | 0.006 | 0.352 | 0.004 | 0.005 | 0.006 | 0.004 | 0.004 | 0.004 | 0.004 | 1 | 0.004 | 0.004 |
