# Supplementary material for: Identification of therapeutic targets applicable to clinical strategies in ovarian cancer
Source: BMC Cancer. 2016 Aug 24;16(1):678. doi: 10.1186/s12885-016-2675-5 (PMC4997769; doi:10.1186/s12885-016-2675-5)
Supplement: Additional file 8: Figure S2. — Dose-effect curves and output from median effect plot for MK1775, BI6727 and Oxozeaenol. (PPT 259 kb) [file 12885_2016_2675_MOESM8_ESM.ppt]

## Slide 1
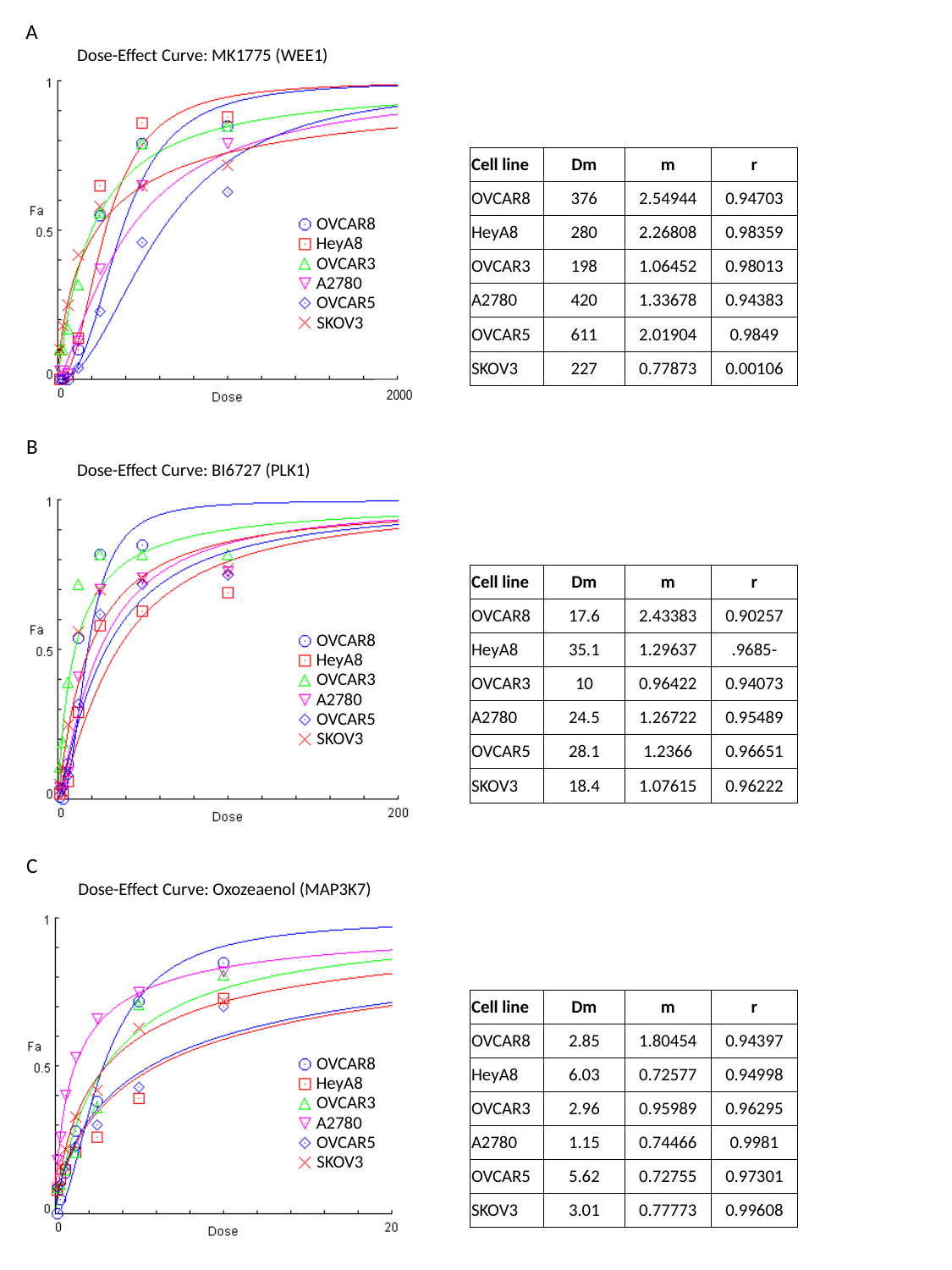

A
Dose-Effect Curve: MK1775 (WEE1)
| Cell line | Dm | m | r |
| --- | --- | --- | --- |
| OVCAR8 | 376 | 2.54944 | 0.94703 |
| HeyA8 | 280 | 2.26808 | 0.98359 |
| OVCAR3 | 198 | 1.06452 | 0.98013 |
| A2780 | 420 | 1.33678 | 0.94383 |
| OVCAR5 | 611 | 2.01904 | 0.9849 |
| SKOV3 | 227 | 0.77873 | 0.00106 |
OVCAR8
HeyA8
OVCAR3
A2780
OVCAR5
SKOV3
2000
B
Dose-Effect Curve: BI6727 (PLK1)
| Cell line | Dm | m | r |
| --- | --- | --- | --- |
| OVCAR8 | 17.6 | 2.43383 | 0.90257 |
| HeyA8 | 35.1 | 1.29637 | .9685- |
| OVCAR3 | 10 | 0.96422 | 0.94073 |
| A2780 | 24.5 | 1.26722 | 0.95489 |
| OVCAR5 | 28.1 | 1.2366 | 0.96651 |
| SKOV3 | 18.4 | 1.07615 | 0.96222 |
OVCAR8
HeyA8
OVCAR3
A2780
OVCAR5
SKOV3
C
Dose-Effect Curve: Oxozeaenol (MAP3K7)
| Cell line | Dm | m | r |
| --- | --- | --- | --- |
| OVCAR8 | 2.85 | 1.80454 | 0.94397 |
| HeyA8 | 6.03 | 0.72577 | 0.94998 |
| OVCAR3 | 2.96 | 0.95989 | 0.96295 |
| A2780 | 1.15 | 0.74466 | 0.9981 |
| OVCAR5 | 5.62 | 0.72755 | 0.97301 |
| SKOV3 | 3.01 | 0.77773 | 0.99608 |
OVCAR8
HeyA8
OVCAR3
A2780
OVCAR5
SKOV3
