# Supplementary material for: Identification of therapeutic targets applicable to clinical strategies in ovarian cancer
Source: BMC Cancer. 2016 Aug 24;16(1):678. doi: 10.1186/s12885-016-2675-5 (PMC4997769; doi:10.1186/s12885-016-2675-5)
Supplement: Additional file 9: Figure S3. — Two-way ANOVA with REGWQ post-hoc test for cisplatin dose effect. (PPTX 65 kb) [file 12885_2016_2675_MOESM9_ESM.pptx]

## Slide 1
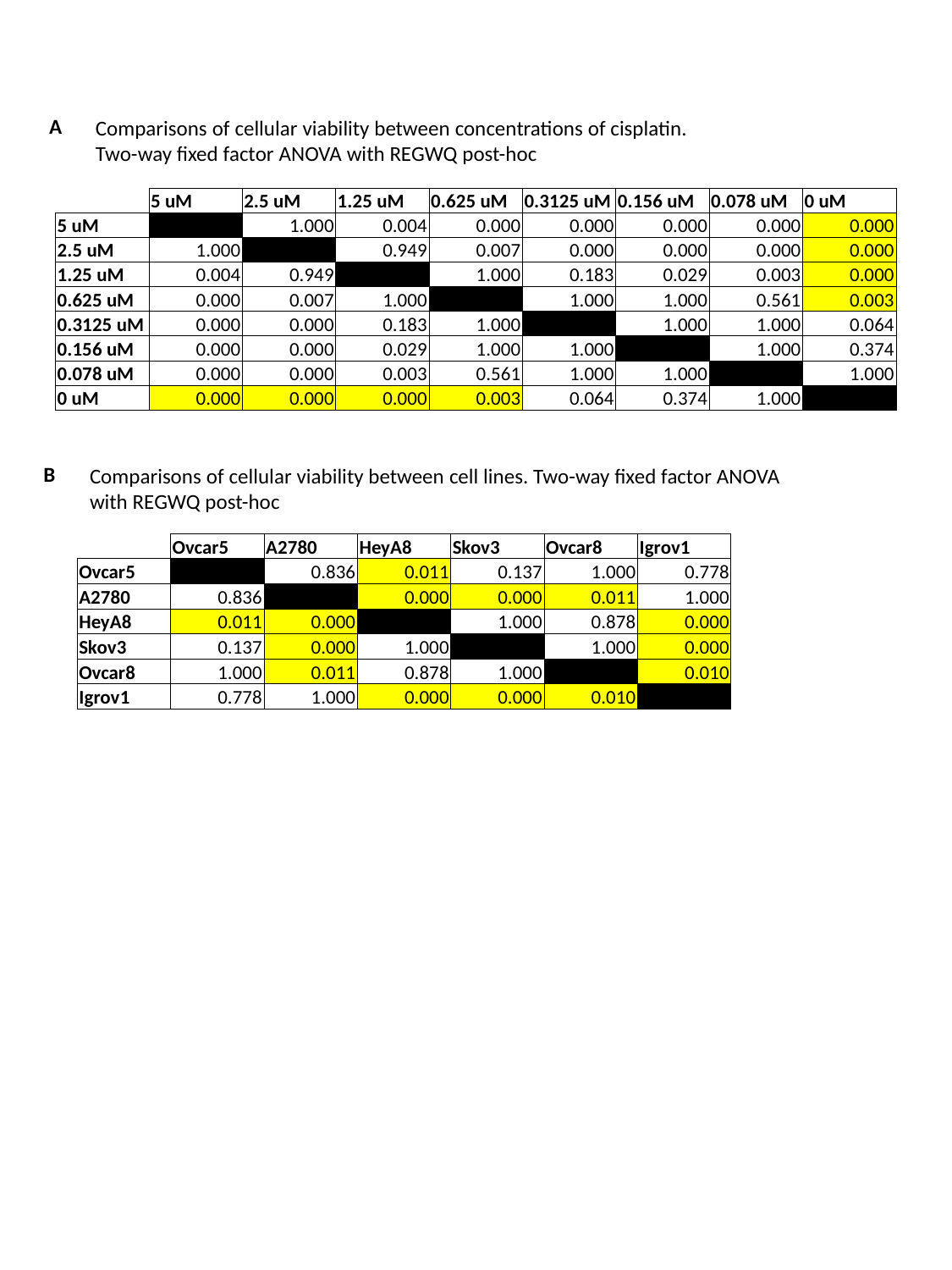

A
Comparisons of cellular viability between concentrations of cisplatin. Two-way fixed factor ANOVA with REGWQ post-hoc
| | 5 uM | 2.5 uM | 1.25 uM | 0.625 uM | 0.3125 uM | 0.156 uM | 0.078 uM | 0 uM |
| --- | --- | --- | --- | --- | --- | --- | --- | --- |
| 5 uM | | 1.000 | 0.004 | 0.000 | 0.000 | 0.000 | 0.000 | 0.000 |
| 2.5 uM | 1.000 | 0.900 | 0.949 | 0.007 | 0.000 | 0.000 | 0.000 | 0.000 |
| 1.25 uM | 0.004 | 0.949 | | 1.000 | 0.183 | 0.029 | 0.003 | 0.000 |
| 0.625 uM | 0.000 | 0.007 | 1.000 | | 1.000 | 1.000 | 0.561 | 0.003 |
| 0.3125 uM | 0.000 | 0.000 | 0.183 | 1.000 | | 1.000 | 1.000 | 0.064 |
| 0.156 uM | 0.000 | 0.000 | 0.029 | 1.000 | 1.000 | | 1.000 | 0.374 |
| 0.078 uM | 0.000 | 0.000 | 0.003 | 0.561 | 1.000 | 1.000 | | 1.000 |
| 0 uM | 0.000 | 0.000 | 0.000 | 0.003 | 0.064 | 0.374 | 1.000 | |
B
Comparisons of cellular viability between cell lines. Two-way fixed factor ANOVA with REGWQ post-hoc
| | Ovcar5 | A2780 | HeyA8 | Skov3 | Ovcar8 | Igrov1 |
| --- | --- | --- | --- | --- | --- | --- |
| Ovcar5 | | 0.836 | 0.011 | 0.137 | 1.000 | 0.778 |
| A2780 | 0.836 | | 0.000 | 0.000 | 0.011 | 1.000 |
| HeyA8 | 0.011 | 0.000 | | 1.000 | 0.878 | 0.000 |
| Skov3 | 0.137 | 0.000 | 1.000 | | 1.000 | 0.000 |
| Ovcar8 | 1.000 | 0.011 | 0.878 | 1.000 | | 0.010 |
| Igrov1 | 0.778 | 1.000 | 0.000 | 0.000 | 0.010 | |
