# Supplementary material for: Identification of therapeutic targets applicable to clinical strategies in ovarian cancer
Source: BMC Cancer. 2016 Aug 24;16(1):678. doi: 10.1186/s12885-016-2675-5 (PMC4997769; doi:10.1186/s12885-016-2675-5)
Supplement: Additional file 10: Figure S4. — Two-way ANOVA with Tukey post-hoc comparing effect of culture conditions and cisplatin or BI6727. (PPTX 112 kb) [file 12885_2016_2675_MOESM10_ESM.pptx]

## Slide 1
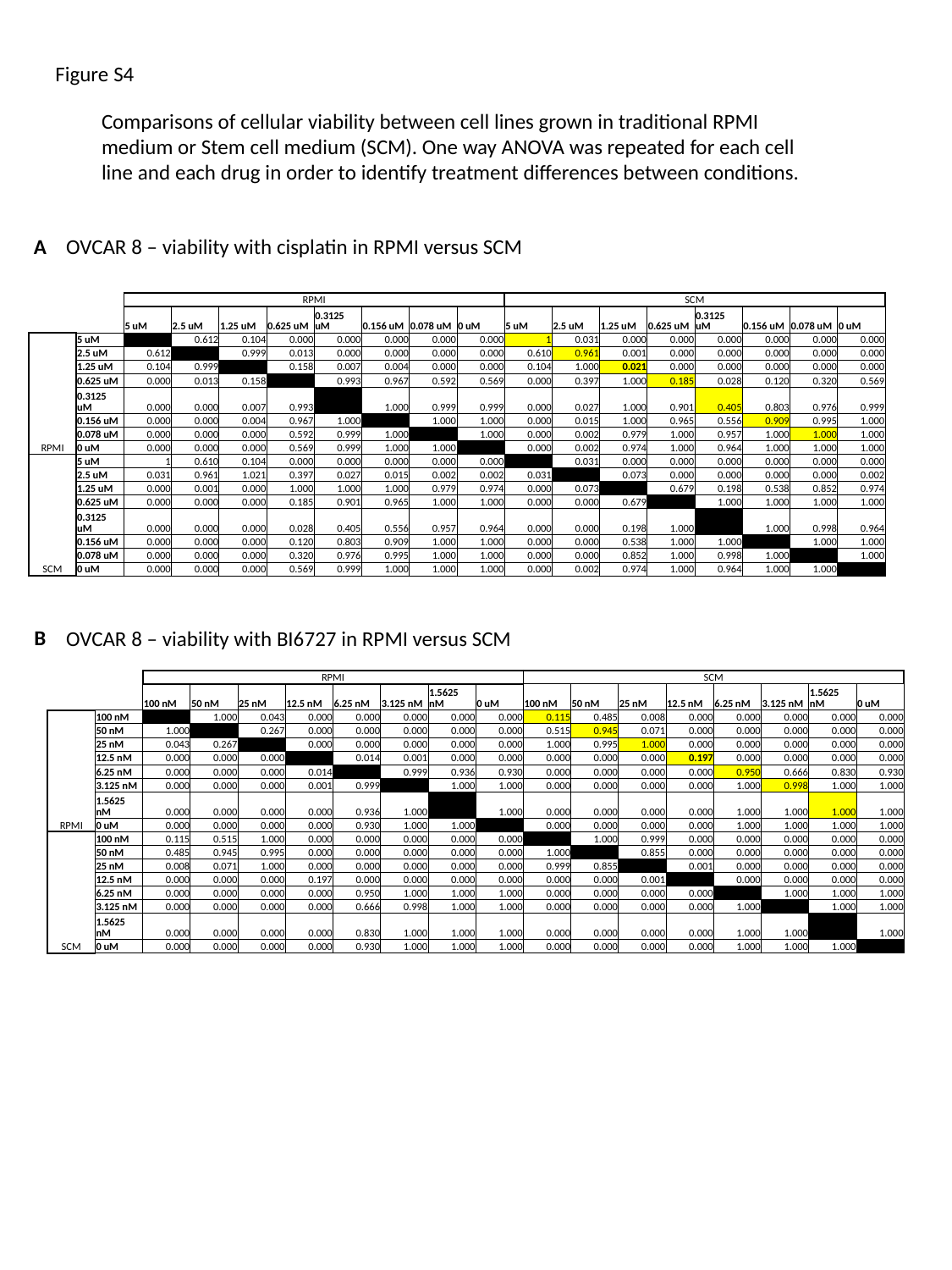

Figure S4
Comparisons of cellular viability between cell lines grown in traditional RPMI medium or Stem cell medium (SCM). One way ANOVA was repeated for each cell line and each drug in order to identify treatment differences between conditions.
A
OVCAR 8 – viability with cisplatin in RPMI versus SCM
| | | RPMI | | | | | | | | SCM | | | | | | | |
| --- | --- | --- | --- | --- | --- | --- | --- | --- | --- | --- | --- | --- | --- | --- | --- | --- | --- |
| | | 5 uM | 2.5 uM | 1.25 uM | 0.625 uM | 0.3125 uM | 0.156 uM | 0.078 uM | 0 uM | 5 uM | 2.5 uM | 1.25 uM | 0.625 uM | 0.3125 uM | 0.156 uM | 0.078 uM | 0 uM |
| RPMI | 5 uM | | 0.612 | 0.104 | 0.000 | 0.000 | 0.000 | 0.000 | 0.000 | 1 | 0.031 | 0.000 | 0.000 | 0.000 | 0.000 | 0.000 | 0.000 |
| | 2.5 uM | 0.612 | | 0.999 | 0.013 | 0.000 | 0.000 | 0.000 | 0.000 | 0.610 | 0.961 | 0.001 | 0.000 | 0.000 | 0.000 | 0.000 | 0.000 |
| | 1.25 uM | 0.104 | 0.999 | | 0.158 | 0.007 | 0.004 | 0.000 | 0.000 | 0.104 | 1.000 | 0.021 | 0.000 | 0.000 | 0.000 | 0.000 | 0.000 |
| | 0.625 uM | 0.000 | 0.013 | 0.158 | | 0.993 | 0.967 | 0.592 | 0.569 | 0.000 | 0.397 | 1.000 | 0.185 | 0.028 | 0.120 | 0.320 | 0.569 |
| | 0.3125 uM | 0.000 | 0.000 | 0.007 | 0.993 | 1.000 | 1.000 | 0.999 | 0.999 | 0.000 | 0.027 | 1.000 | 0.901 | 0.405 | 0.803 | 0.976 | 0.999 |
| | 0.156 uM | 0.000 | 0.000 | 0.004 | 0.967 | 1.000 | | 1.000 | 1.000 | 0.000 | 0.015 | 1.000 | 0.965 | 0.556 | 0.909 | 0.995 | 1.000 |
| | 0.078 uM | 0.000 | 0.000 | 0.000 | 0.592 | 0.999 | 1.000 | | 1.000 | 0.000 | 0.002 | 0.979 | 1.000 | 0.957 | 1.000 | 1.000 | 1.000 |
| | 0 uM | 0.000 | 0.000 | 0.000 | 0.569 | 0.999 | 1.000 | 1.000 | | 0.000 | 0.002 | 0.974 | 1.000 | 0.964 | 1.000 | 1.000 | 1.000 |
| SCM | 5 uM | 1 | 0.610 | 0.104 | 0.000 | 0.000 | 0.000 | 0.000 | 0.000 | 1 | 0.031 | 0.000 | 0.000 | 0.000 | 0.000 | 0.000 | 0.000 |
| | 2.5 uM | 0.031 | 0.961 | 1.021 | 0.397 | 0.027 | 0.015 | 0.002 | 0.002 | 0.031 | | 0.073 | 0.000 | 0.000 | 0.000 | 0.000 | 0.002 |
| | 1.25 uM | 0.000 | 0.001 | 0.000 | 1.000 | 1.000 | 1.000 | 0.979 | 0.974 | 0.000 | 0.073 | | 0.679 | 0.198 | 0.538 | 0.852 | 0.974 |
| | 0.625 uM | 0.000 | 0.000 | 0.000 | 0.185 | 0.901 | 0.965 | 1.000 | 1.000 | 0.000 | 0.000 | 0.679 | 1.000 | 1.000 | 1.000 | 1.000 | 1.000 |
| | 0.3125 uM | 0.000 | 0.000 | 0.000 | 0.028 | 0.405 | 0.556 | 0.957 | 0.964 | 0.000 | 0.000 | 0.198 | 1.000 | | 1.000 | 0.998 | 0.964 |
| | 0.156 uM | 0.000 | 0.000 | 0.000 | 0.120 | 0.803 | 0.909 | 1.000 | 1.000 | 0.000 | 0.000 | 0.538 | 1.000 | 1.000 | | 1.000 | 1.000 |
| | 0.078 uM | 0.000 | 0.000 | 0.000 | 0.320 | 0.976 | 0.995 | 1.000 | 1.000 | 0.000 | 0.000 | 0.852 | 1.000 | 0.998 | 1.000 | | 1.000 |
| | 0 uM | 0.000 | 0.000 | 0.000 | 0.569 | 0.999 | 1.000 | 1.000 | 1.000 | 0.000 | 0.002 | 0.974 | 1.000 | 0.964 | 1.000 | 1.000 | |
B
OVCAR 8 – viability with BI6727 in RPMI versus SCM
| | | RPMI | | | | | | | | SCM | | | | | | | |
| --- | --- | --- | --- | --- | --- | --- | --- | --- | --- | --- | --- | --- | --- | --- | --- | --- | --- |
| | | 100 nM | 50 nM | 25 nM | 12.5 nM | 6.25 nM | 3.125 nM | 1.5625 nM | 0 uM | 100 nM | 50 nM | 25 nM | 12.5 nM | 6.25 nM | 3.125 nM | 1.5625 nM | 0 uM |
| RPMI | 100 nM | | 1.000 | 0.043 | 0.000 | 0.000 | 0.000 | 0.000 | 0.000 | 0.115 | 0.485 | 0.008 | 0.000 | 0.000 | 0.000 | 0.000 | 0.000 |
| | 50 nM | 1.000 | | 0.267 | 0.000 | 0.000 | 0.000 | 0.000 | 0.000 | 0.515 | 0.945 | 0.071 | 0.000 | 0.000 | 0.000 | 0.000 | 0.000 |
| | 25 nM | 0.043 | 0.267 | | 0.000 | 0.000 | 0.000 | 0.000 | 0.000 | 1.000 | 0.995 | 1.000 | 0.000 | 0.000 | 0.000 | 0.000 | 0.000 |
| | 12.5 nM | 0.000 | 0.000 | 0.000 | | 0.014 | 0.001 | 0.000 | 0.000 | 0.000 | 0.000 | 0.000 | 0.197 | 0.000 | 0.000 | 0.000 | 0.000 |
| | 6.25 nM | 0.000 | 0.000 | 0.000 | 0.014 | | 0.999 | 0.936 | 0.930 | 0.000 | 0.000 | 0.000 | 0.000 | 0.950 | 0.666 | 0.830 | 0.930 |
| | 3.125 nM | 0.000 | 0.000 | 0.000 | 0.001 | 0.999 | 1.000 | 1.000 | 1.000 | 0.000 | 0.000 | 0.000 | 0.000 | 1.000 | 0.998 | 1.000 | 1.000 |
| | 1.5625 nM | 0.000 | 0.000 | 0.000 | 0.000 | 0.936 | 1.000 | | 1.000 | 0.000 | 0.000 | 0.000 | 0.000 | 1.000 | 1.000 | 1.000 | 1.000 |
| | 0 uM | 0.000 | 0.000 | 0.000 | 0.000 | 0.930 | 1.000 | 1.000 | | 0.000 | 0.000 | 0.000 | 0.000 | 1.000 | 1.000 | 1.000 | 1.000 |
| SCM | 100 nM | 0.115 | 0.515 | 1.000 | 0.000 | 0.000 | 0.000 | 0.000 | 0.000 | | 1.000 | 0.999 | 0.000 | 0.000 | 0.000 | 0.000 | 0.000 |
| | 50 nM | 0.485 | 0.945 | 0.995 | 0.000 | 0.000 | 0.000 | 0.000 | 0.000 | 1.000 | | 0.855 | 0.000 | 0.000 | 0.000 | 0.000 | 0.000 |
| | 25 nM | 0.008 | 0.071 | 1.000 | 0.000 | 0.000 | 0.000 | 0.000 | 0.000 | 0.999 | 0.855 | | 0.001 | 0.000 | 0.000 | 0.000 | 0.000 |
| | 12.5 nM | 0.000 | 0.000 | 0.000 | 0.197 | 0.000 | 0.000 | 0.000 | 0.000 | 0.000 | 0.000 | 0.001 | | 0.000 | 0.000 | 0.000 | 0.000 |
| | 6.25 nM | 0.000 | 0.000 | 0.000 | 0.000 | 0.950 | 1.000 | 1.000 | 1.000 | 0.000 | 0.000 | 0.000 | 0.000 | | 1.000 | 1.000 | 1.000 |
| | 3.125 nM | 0.000 | 0.000 | 0.000 | 0.000 | 0.666 | 0.998 | 1.000 | 1.000 | 0.000 | 0.000 | 0.000 | 0.000 | 1.000 | | 1.000 | 1.000 |
| | 1.5625 nM | 0.000 | 0.000 | 0.000 | 0.000 | 0.830 | 1.000 | 1.000 | 1.000 | 0.000 | 0.000 | 0.000 | 0.000 | 1.000 | 1.000 | | 1.000 |
| | 0 uM | 0.000 | 0.000 | 0.000 | 0.000 | 0.930 | 1.000 | 1.000 | 1.000 | 0.000 | 0.000 | 0.000 | 0.000 | 1.000 | 1.000 | 1.000 | |

## Slide 2
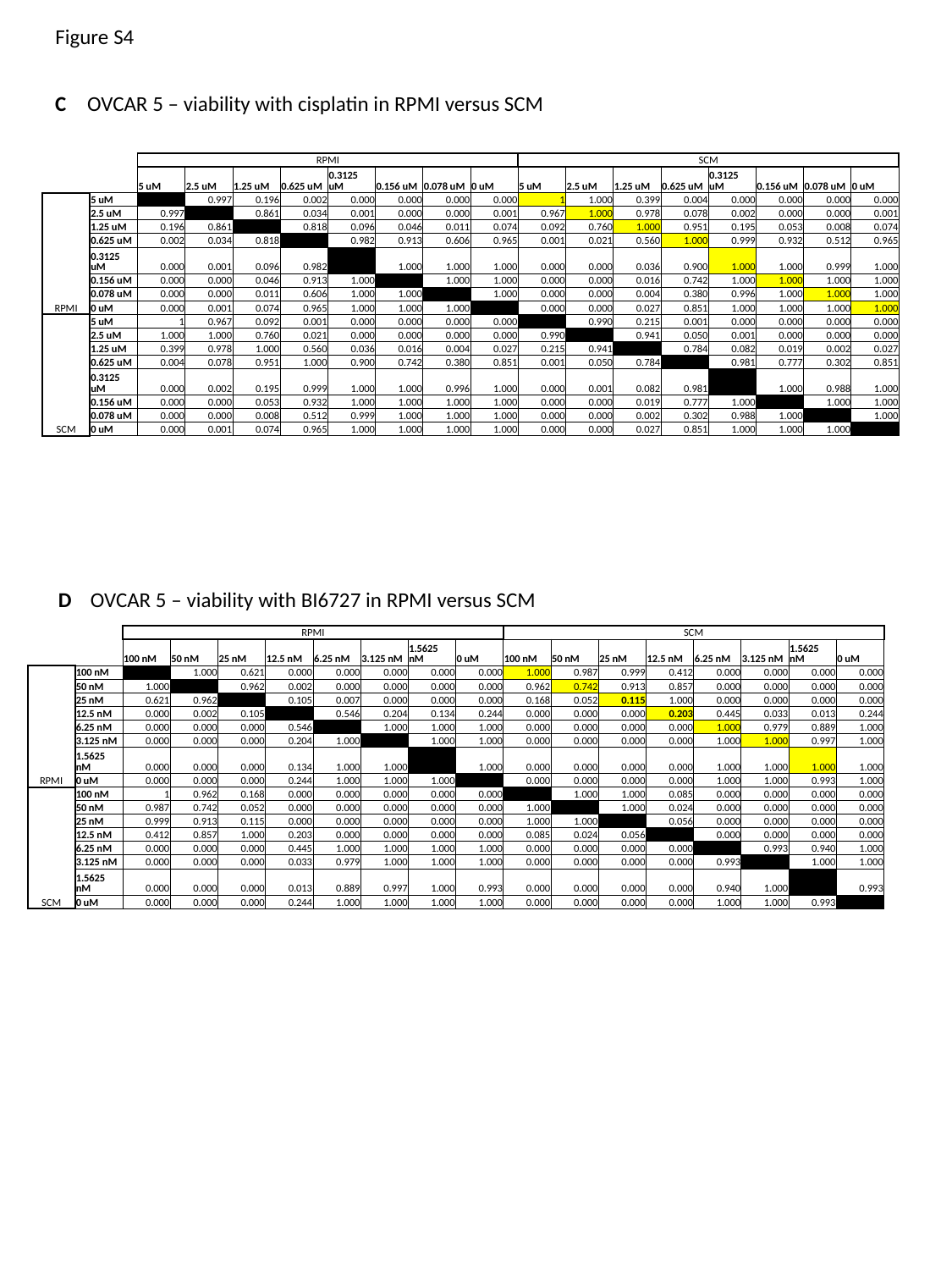

Figure S4
C
OVCAR 5 – viability with cisplatin in RPMI versus SCM
| | | RPMI | | | | | | | | SCM | | | | | | | |
| --- | --- | --- | --- | --- | --- | --- | --- | --- | --- | --- | --- | --- | --- | --- | --- | --- | --- |
| | | 5 uM | 2.5 uM | 1.25 uM | 0.625 uM | 0.3125 uM | 0.156 uM | 0.078 uM | 0 uM | 5 uM | 2.5 uM | 1.25 uM | 0.625 uM | 0.3125 uM | 0.156 uM | 0.078 uM | 0 uM |
| RPMI | 5 uM | | 0.997 | 0.196 | 0.002 | 0.000 | 0.000 | 0.000 | 0.000 | 1 | 1.000 | 0.399 | 0.004 | 0.000 | 0.000 | 0.000 | 0.000 |
| | 2.5 uM | 0.997 | | 0.861 | 0.034 | 0.001 | 0.000 | 0.000 | 0.001 | 0.967 | 1.000 | 0.978 | 0.078 | 0.002 | 0.000 | 0.000 | 0.001 |
| | 1.25 uM | 0.196 | 0.861 | 0.934 | 0.818 | 0.096 | 0.046 | 0.011 | 0.074 | 0.092 | 0.760 | 1.000 | 0.951 | 0.195 | 0.053 | 0.008 | 0.074 |
| | 0.625 uM | 0.002 | 0.034 | 0.818 | | 0.982 | 0.913 | 0.606 | 0.965 | 0.001 | 0.021 | 0.560 | 1.000 | 0.999 | 0.932 | 0.512 | 0.965 |
| | 0.3125 uM | 0.000 | 0.001 | 0.096 | 0.982 | | 1.000 | 1.000 | 1.000 | 0.000 | 0.000 | 0.036 | 0.900 | 1.000 | 1.000 | 0.999 | 1.000 |
| | 0.156 uM | 0.000 | 0.000 | 0.046 | 0.913 | 1.000 | 1.000 | 1.000 | 1.000 | 0.000 | 0.000 | 0.016 | 0.742 | 1.000 | 1.000 | 1.000 | 1.000 |
| | 0.078 uM | 0.000 | 0.000 | 0.011 | 0.606 | 1.000 | 1.000 | | 1.000 | 0.000 | 0.000 | 0.004 | 0.380 | 0.996 | 1.000 | 1.000 | 1.000 |
| | 0 uM | 0.000 | 0.001 | 0.074 | 0.965 | 1.000 | 1.000 | 1.000 | | 0.000 | 0.000 | 0.027 | 0.851 | 1.000 | 1.000 | 1.000 | 1.000 |
| SCM | 5 uM | 1 | 0.967 | 0.092 | 0.001 | 0.000 | 0.000 | 0.000 | 0.000 | 0.99 | 0.990 | 0.215 | 0.001 | 0.000 | 0.000 | 0.000 | 0.000 |
| | 2.5 uM | 1.000 | 1.000 | 0.760 | 0.021 | 0.000 | 0.000 | 0.000 | 0.000 | 0.990 | | 0.941 | 0.050 | 0.001 | 0.000 | 0.000 | 0.000 |
| | 1.25 uM | 0.399 | 0.978 | 1.000 | 0.560 | 0.036 | 0.016 | 0.004 | 0.027 | 0.215 | 0.941 | 1.000 | 0.784 | 0.082 | 0.019 | 0.002 | 0.027 |
| | 0.625 uM | 0.004 | 0.078 | 0.951 | 1.000 | 0.900 | 0.742 | 0.380 | 0.851 | 0.001 | 0.050 | 0.784 | | 0.981 | 0.777 | 0.302 | 0.851 |
| | 0.3125 uM | 0.000 | 0.002 | 0.195 | 0.999 | 1.000 | 1.000 | 0.996 | 1.000 | 0.000 | 0.001 | 0.082 | 0.981 | 1.000 | 1.000 | 0.988 | 1.000 |
| | 0.156 uM | 0.000 | 0.000 | 0.053 | 0.932 | 1.000 | 1.000 | 1.000 | 1.000 | 0.000 | 0.000 | 0.019 | 0.777 | 1.000 | 1.000 | 1.000 | 1.000 |
| | 0.078 uM | 0.000 | 0.000 | 0.008 | 0.512 | 0.999 | 1.000 | 1.000 | 1.000 | 0.000 | 0.000 | 0.002 | 0.302 | 0.988 | 1.000 | 1.000 | 1.000 |
| | 0 uM | 0.000 | 0.001 | 0.074 | 0.965 | 1.000 | 1.000 | 1.000 | 1.000 | 0.000 | 0.000 | 0.027 | 0.851 | 1.000 | 1.000 | 1.000 | 1.000 |
D
OVCAR 5 – viability with BI6727 in RPMI versus SCM
| | | RPMI | | | | | | | | SCM | | | | | | | |
| --- | --- | --- | --- | --- | --- | --- | --- | --- | --- | --- | --- | --- | --- | --- | --- | --- | --- |
| | | 100 nM | 50 nM | 25 nM | 12.5 nM | 6.25 nM | 3.125 nM | 1.5625 nM | 0 uM | 100 nM | 50 nM | 25 nM | 12.5 nM | 6.25 nM | 3.125 nM | 1.5625 nM | 0 uM |
| RPMI | 100 nM | | 1.000 | 0.621 | 0.000 | 0.000 | 0.000 | 0.000 | 0.000 | 1.000 | 0.987 | 0.999 | 0.412 | 0.000 | 0.000 | 0.000 | 0.000 |
| | 50 nM | 1.000 | | 0.962 | 0.002 | 0.000 | 0.000 | 0.000 | 0.000 | 0.962 | 0.742 | 0.913 | 0.857 | 0.000 | 0.000 | 0.000 | 0.000 |
| | 25 nM | 0.621 | 0.962 | | 0.105 | 0.007 | 0.000 | 0.000 | 0.000 | 0.168 | 0.052 | 0.115 | 1.000 | 0.000 | 0.000 | 0.000 | 0.000 |
| | 12.5 nM | 0.000 | 0.002 | 0.105 | | 0.546 | 0.204 | 0.134 | 0.244 | 0.000 | 0.000 | 0.000 | 0.203 | 0.445 | 0.033 | 0.013 | 0.244 |
| | 6.25 nM | 0.000 | 0.000 | 0.000 | 0.546 | | 1.000 | 1.000 | 1.000 | 0.000 | 0.000 | 0.000 | 0.000 | 1.000 | 0.979 | 0.889 | 1.000 |
| | 3.125 nM | 0.000 | 0.000 | 0.000 | 0.204 | 1.000 | | 1.000 | 1.000 | 0.000 | 0.000 | 0.000 | 0.000 | 1.000 | 1.000 | 0.997 | 1.000 |
| | 1.5625 nM | 0.000 | 0.000 | 0.000 | 0.134 | 1.000 | 1.000 | | 1.000 | 0.000 | 0.000 | 0.000 | 0.000 | 1.000 | 1.000 | 1.000 | 1.000 |
| | 0 uM | 0.000 | 0.000 | 0.000 | 0.244 | 1.000 | 1.000 | 1.000 | | 0.000 | 0.000 | 0.000 | 0.000 | 1.000 | 1.000 | 0.993 | 1.000 |
| SCM | 100 nM | 1 | 0.962 | 0.168 | 0.000 | 0.000 | 0.000 | 0.000 | 0.000 | | 1.000 | 1.000 | 0.085 | 0.000 | 0.000 | 0.000 | 0.000 |
| | 50 nM | 0.987 | 0.742 | 0.052 | 0.000 | 0.000 | 0.000 | 0.000 | 0.000 | 1.000 | | 1.000 | 0.024 | 0.000 | 0.000 | 0.000 | 0.000 |
| | 25 nM | 0.999 | 0.913 | 0.115 | 0.000 | 0.000 | 0.000 | 0.000 | 0.000 | 1.000 | 1.000 | | 0.056 | 0.000 | 0.000 | 0.000 | 0.000 |
| | 12.5 nM | 0.412 | 0.857 | 1.000 | 0.203 | 0.000 | 0.000 | 0.000 | 0.000 | 0.085 | 0.024 | 0.056 | | 0.000 | 0.000 | 0.000 | 0.000 |
| | 6.25 nM | 0.000 | 0.000 | 0.000 | 0.445 | 1.000 | 1.000 | 1.000 | 1.000 | 0.000 | 0.000 | 0.000 | 0.000 | | 0.993 | 0.940 | 1.000 |
| | 3.125 nM | 0.000 | 0.000 | 0.000 | 0.033 | 0.979 | 1.000 | 1.000 | 1.000 | 0.000 | 0.000 | 0.000 | 0.000 | 0.993 | | 1.000 | 1.000 |
| | 1.5625 nM | 0.000 | 0.000 | 0.000 | 0.013 | 0.889 | 0.997 | 1.000 | 0.993 | 0.000 | 0.000 | 0.000 | 0.000 | 0.940 | 1.000 | | 0.993 |
| | 0 uM | 0.000 | 0.000 | 0.000 | 0.244 | 1.000 | 1.000 | 1.000 | 1.000 | 0.000 | 0.000 | 0.000 | 0.000 | 1.000 | 1.000 | 0.993 | |
