# Supplementary material for: Identification of therapeutic targets applicable to clinical strategies in ovarian cancer
Source: BMC Cancer. 2016 Aug 24;16(1):678. doi: 10.1186/s12885-016-2675-5 (PMC4997769; doi:10.1186/s12885-016-2675-5)
Supplement: Additional file 11: Figure S5. — Two-way ANOVA with Dunnett post-hoc comparing sequential exposure to BI6727 and MK1775. (PPTX 70 kb) [file 12885_2016_2675_MOESM11_ESM.pptx]

## Slide 1
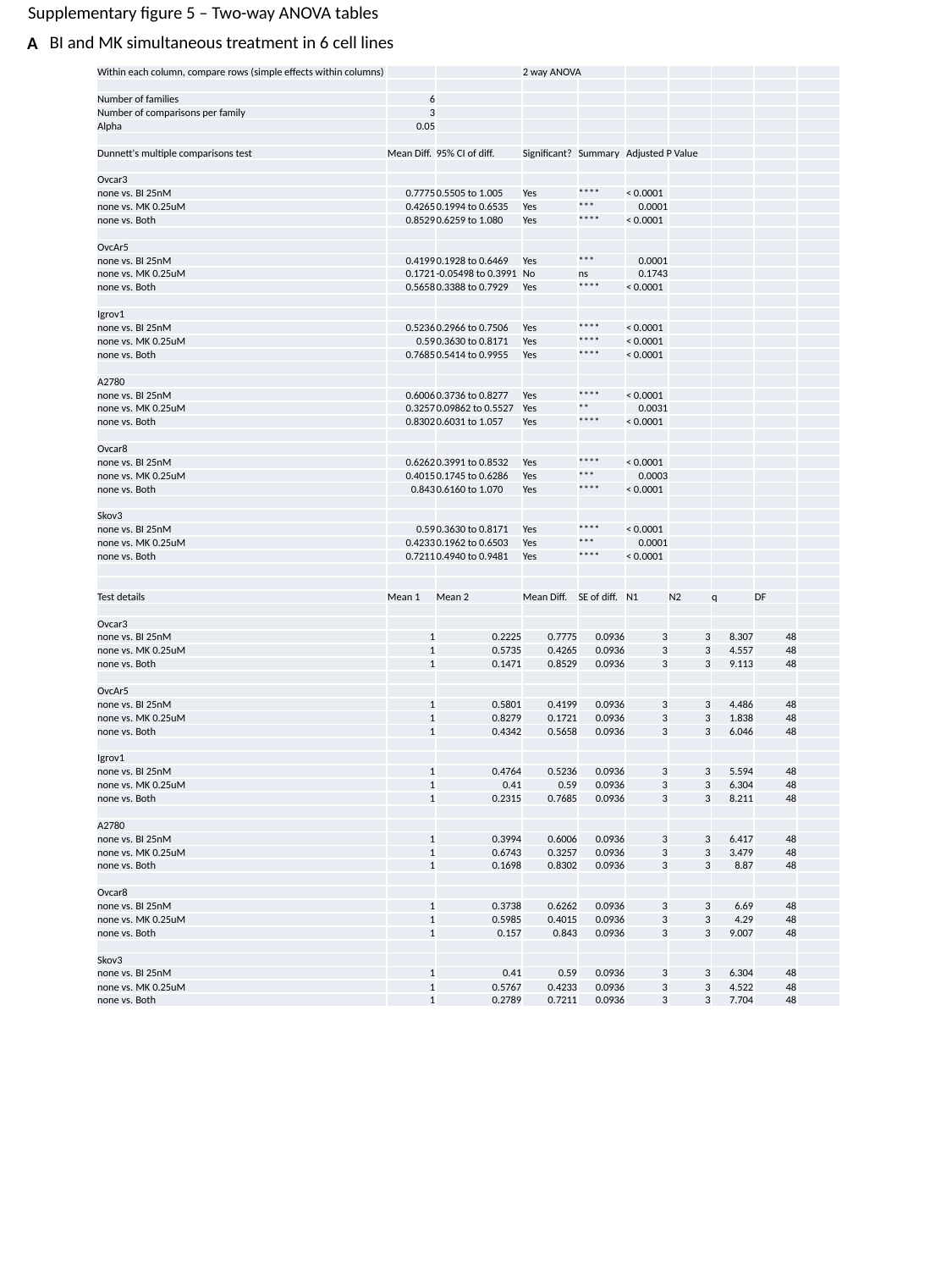

Supplementary figure 5 – Two-way ANOVA tables
BI and MK simultaneous treatment in 6 cell lines
A
| Within each column, compare rows (simple effects within columns) | | | 2 way ANOVA | | | | | | |
| --- | --- | --- | --- | --- | --- | --- | --- | --- | --- |
| | | | | | | | | | |
| Number of families | 6 | | | | | | | | |
| Number of comparisons per family | 3 | | | | | | | | |
| Alpha | 0.05 | | | | | | | | |
| | | | | | | | | | |
| Dunnett's multiple comparisons test | Mean Diff. | 95% CI of diff. | Significant? | Summary | Adjusted P Value | | | | |
| | | | | | | | | | |
| Ovcar3 | | | | | | | | | |
| none vs. BI 25nM | 0.7775 | 0.5505 to 1.005 | Yes | \*\*\*\* | < 0.0001 | | | | |
| none vs. MK 0.25uM | 0.4265 | 0.1994 to 0.6535 | Yes | \*\*\* | 0.0001 | | | | |
| none vs. Both | 0.8529 | 0.6259 to 1.080 | Yes | \*\*\*\* | < 0.0001 | | | | |
| | | | | | | | | | |
| OvcAr5 | | | | | | | | | |
| none vs. BI 25nM | 0.4199 | 0.1928 to 0.6469 | Yes | \*\*\* | 0.0001 | | | | |
| none vs. MK 0.25uM | 0.1721 | -0.05498 to 0.3991 | No | ns | 0.1743 | | | | |
| none vs. Both | 0.5658 | 0.3388 to 0.7929 | Yes | \*\*\*\* | < 0.0001 | | | | |
| | | | | | | | | | |
| Igrov1 | | | | | | | | | |
| none vs. BI 25nM | 0.5236 | 0.2966 to 0.7506 | Yes | \*\*\*\* | < 0.0001 | | | | |
| none vs. MK 0.25uM | 0.59 | 0.3630 to 0.8171 | Yes | \*\*\*\* | < 0.0001 | | | | |
| none vs. Both | 0.7685 | 0.5414 to 0.9955 | Yes | \*\*\*\* | < 0.0001 | | | | |
| | | | | | | | | | |
| A2780 | | | | | | | | | |
| none vs. BI 25nM | 0.6006 | 0.3736 to 0.8277 | Yes | \*\*\*\* | < 0.0001 | | | | |
| none vs. MK 0.25uM | 0.3257 | 0.09862 to 0.5527 | Yes | \*\* | 0.0031 | | | | |
| none vs. Both | 0.8302 | 0.6031 to 1.057 | Yes | \*\*\*\* | < 0.0001 | | | | |
| | | | | | | | | | |
| Ovcar8 | | | | | | | | | |
| none vs. BI 25nM | 0.6262 | 0.3991 to 0.8532 | Yes | \*\*\*\* | < 0.0001 | | | | |
| none vs. MK 0.25uM | 0.4015 | 0.1745 to 0.6286 | Yes | \*\*\* | 0.0003 | | | | |
| none vs. Both | 0.843 | 0.6160 to 1.070 | Yes | \*\*\*\* | < 0.0001 | | | | |
| | | | | | | | | | |
| Skov3 | | | | | | | | | |
| none vs. BI 25nM | 0.59 | 0.3630 to 0.8171 | Yes | \*\*\*\* | < 0.0001 | | | | |
| none vs. MK 0.25uM | 0.4233 | 0.1962 to 0.6503 | Yes | \*\*\* | 0.0001 | | | | |
| none vs. Both | 0.7211 | 0.4940 to 0.9481 | Yes | \*\*\*\* | < 0.0001 | | | | |
| | | | | | | | | | |
| | | | | | | | | | |
| Test details | Mean 1 | Mean 2 | Mean Diff. | SE of diff. | N1 | N2 | q | DF | |
| | | | | | | | | | |
| Ovcar3 | | | | | | | | | |
| none vs. BI 25nM | 1 | 0.2225 | 0.7775 | 0.0936 | 3 | 3 | 8.307 | 48 | |
| none vs. MK 0.25uM | 1 | 0.5735 | 0.4265 | 0.0936 | 3 | 3 | 4.557 | 48 | |
| none vs. Both | 1 | 0.1471 | 0.8529 | 0.0936 | 3 | 3 | 9.113 | 48 | |
| | | | | | | | | | |
| OvcAr5 | | | | | | | | | |
| none vs. BI 25nM | 1 | 0.5801 | 0.4199 | 0.0936 | 3 | 3 | 4.486 | 48 | |
| none vs. MK 0.25uM | 1 | 0.8279 | 0.1721 | 0.0936 | 3 | 3 | 1.838 | 48 | |
| none vs. Both | 1 | 0.4342 | 0.5658 | 0.0936 | 3 | 3 | 6.046 | 48 | |
| | | | | | | | | | |
| Igrov1 | | | | | | | | | |
| none vs. BI 25nM | 1 | 0.4764 | 0.5236 | 0.0936 | 3 | 3 | 5.594 | 48 | |
| none vs. MK 0.25uM | 1 | 0.41 | 0.59 | 0.0936 | 3 | 3 | 6.304 | 48 | |
| none vs. Both | 1 | 0.2315 | 0.7685 | 0.0936 | 3 | 3 | 8.211 | 48 | |
| | | | | | | | | | |
| A2780 | | | | | | | | | |
| none vs. BI 25nM | 1 | 0.3994 | 0.6006 | 0.0936 | 3 | 3 | 6.417 | 48 | |
| none vs. MK 0.25uM | 1 | 0.6743 | 0.3257 | 0.0936 | 3 | 3 | 3.479 | 48 | |
| none vs. Both | 1 | 0.1698 | 0.8302 | 0.0936 | 3 | 3 | 8.87 | 48 | |
| | | | | | | | | | |
| Ovcar8 | | | | | | | | | |
| none vs. BI 25nM | 1 | 0.3738 | 0.6262 | 0.0936 | 3 | 3 | 6.69 | 48 | |
| none vs. MK 0.25uM | 1 | 0.5985 | 0.4015 | 0.0936 | 3 | 3 | 4.29 | 48 | |
| none vs. Both | 1 | 0.157 | 0.843 | 0.0936 | 3 | 3 | 9.007 | 48 | |
| | | | | | | | | | |
| Skov3 | | | | | | | | | |
| none vs. BI 25nM | 1 | 0.41 | 0.59 | 0.0936 | 3 | 3 | 6.304 | 48 | |
| none vs. MK 0.25uM | 1 | 0.5767 | 0.4233 | 0.0936 | 3 | 3 | 4.522 | 48 | |
| none vs. Both | 1 | 0.2789 | 0.7211 | 0.0936 | 3 | 3 | 7.704 | 48 | |

## Slide 2
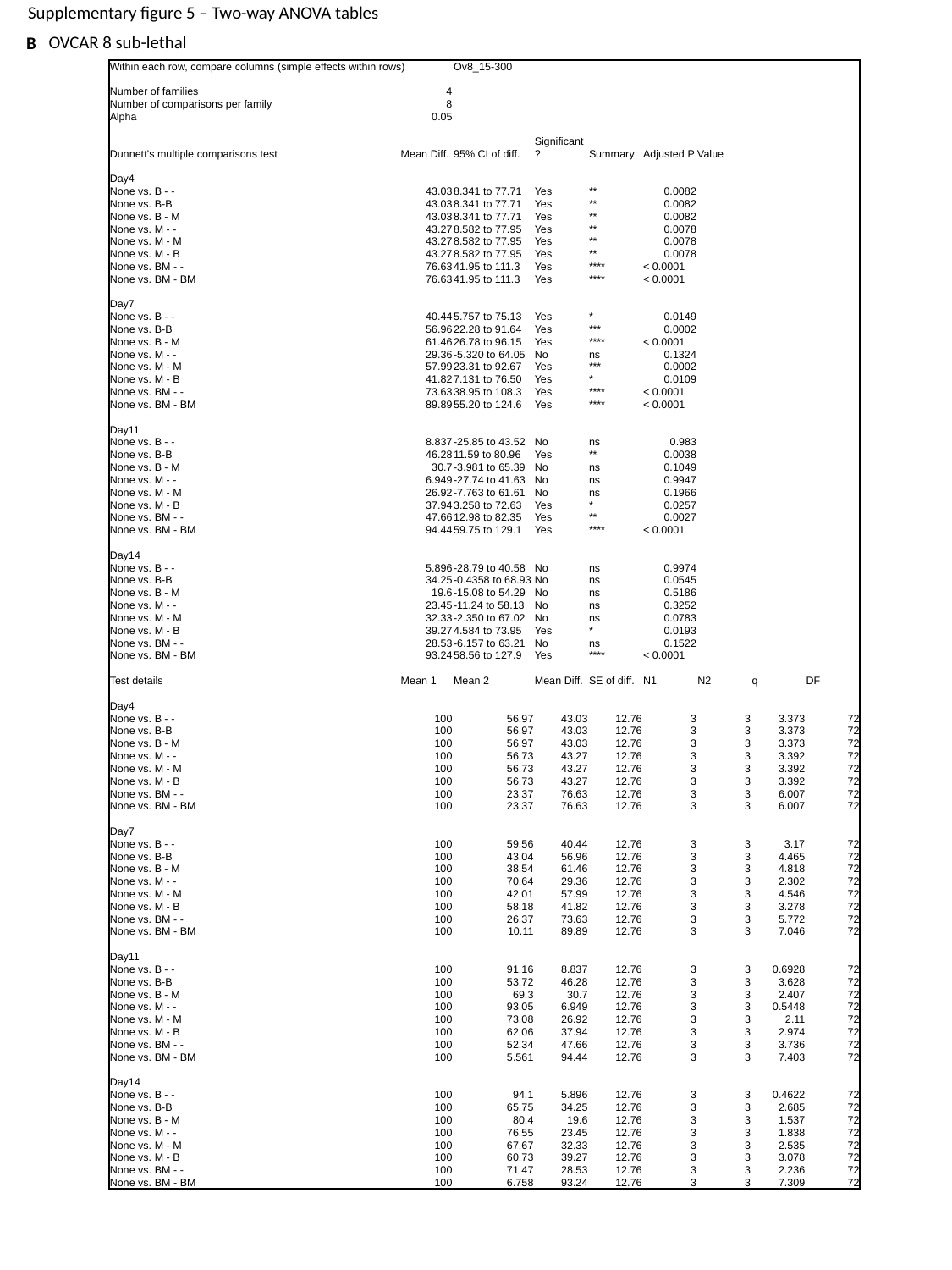

Supplementary figure 5 – Two-way ANOVA tables
OVCAR 8 sub-lethal
B
| Within each row, compare columns (simple effects within rows) | | Ov8\_15-300 | | | | | | |
| --- | --- | --- | --- | --- | --- | --- | --- | --- |
| | | | | | | | | |
| Number of families | 4 | | | | | | | |
| Number of comparisons per family | 8 | | | | | | | |
| Alpha | 0.05 | | | | | | | |
| | | | | | | | | |
| Dunnett's multiple comparisons test | Mean Diff. | 95% CI of diff. | Significant? | Summary | Adjusted P Value | | | |
| | | | | | | | | |
| Day4 | | | | | | | | |
| None vs. B - - | 43.03 | 8.341 to 77.71 | Yes | \*\* | 0.0082 | | | |
| None vs. B-B | 43.03 | 8.341 to 77.71 | Yes | \*\* | 0.0082 | | | |
| None vs. B - M | 43.03 | 8.341 to 77.71 | Yes | \*\* | 0.0082 | | | |
| None vs. M - - | 43.27 | 8.582 to 77.95 | Yes | \*\* | 0.0078 | | | |
| None vs. M - M | 43.27 | 8.582 to 77.95 | Yes | \*\* | 0.0078 | | | |
| None vs. M - B | 43.27 | 8.582 to 77.95 | Yes | \*\* | 0.0078 | | | |
| None vs. BM - - | 76.63 | 41.95 to 111.3 | Yes | \*\*\*\* | < 0.0001 | | | |
| None vs. BM - BM | 76.63 | 41.95 to 111.3 | Yes | \*\*\*\* | < 0.0001 | | | |
| | | | | | | | | |
| Day7 | | | | | | | | |
| None vs. B - - | 40.44 | 5.757 to 75.13 | Yes | \* | 0.0149 | | | |
| None vs. B-B | 56.96 | 22.28 to 91.64 | Yes | \*\*\* | 0.0002 | | | |
| None vs. B - M | 61.46 | 26.78 to 96.15 | Yes | \*\*\*\* | < 0.0001 | | | |
| None vs. M - - | 29.36 | -5.320 to 64.05 | No | ns | 0.1324 | | | |
| None vs. M - M | 57.99 | 23.31 to 92.67 | Yes | \*\*\* | 0.0002 | | | |
| None vs. M - B | 41.82 | 7.131 to 76.50 | Yes | \* | 0.0109 | | | |
| None vs. BM - - | 73.63 | 38.95 to 108.3 | Yes | \*\*\*\* | < 0.0001 | | | |
| None vs. BM - BM | 89.89 | 55.20 to 124.6 | Yes | \*\*\*\* | < 0.0001 | | | |
| | | | | | | | | |
| Day11 | | | | | | | | |
| None vs. B - - | 8.837 | -25.85 to 43.52 | No | ns | 0.983 | | | |
| None vs. B-B | 46.28 | 11.59 to 80.96 | Yes | \*\* | 0.0038 | | | |
| None vs. B - M | 30.7 | -3.981 to 65.39 | No | ns | 0.1049 | | | |
| None vs. M - - | 6.949 | -27.74 to 41.63 | No | ns | 0.9947 | | | |
| None vs. M - M | 26.92 | -7.763 to 61.61 | No | ns | 0.1966 | | | |
| None vs. M - B | 37.94 | 3.258 to 72.63 | Yes | \* | 0.0257 | | | |
| None vs. BM - - | 47.66 | 12.98 to 82.35 | Yes | \*\* | 0.0027 | | | |
| None vs. BM - BM | 94.44 | 59.75 to 129.1 | Yes | \*\*\*\* | < 0.0001 | | | |
| | | | | | | | | |
| Day14 | | | | | | | | |
| None vs. B - - | 5.896 | -28.79 to 40.58 | No | ns | 0.9974 | | | |
| None vs. B-B | 34.25 | -0.4358 to 68.93 | No | ns | 0.0545 | | | |
| None vs. B - M | 19.6 | -15.08 to 54.29 | No | ns | 0.5186 | | | |
| None vs. M - - | 23.45 | -11.24 to 58.13 | No | ns | 0.3252 | | | |
| None vs. M - M | 32.33 | -2.350 to 67.02 | No | ns | 0.0783 | | | |
| None vs. M - B | 39.27 | 4.584 to 73.95 | Yes | \* | 0.0193 | | | |
| None vs. BM - - | 28.53 | -6.157 to 63.21 | No | ns | 0.1522 | | | |
| None vs. BM - BM | 93.24 | 58.56 to 127.9 | Yes | \*\*\*\* | < 0.0001 | | | |
| | | | | | | | | |
| Test details | Mean 1 | Mean 2 | Mean Diff. | SE of diff. | N1 | N2 | q | DF |
| | | | | | | | | |
| Day4 | | | | | | | | |
| None vs. B - - | 100 | 56.97 | 43.03 | 12.76 | 3 | 3 | 3.373 | 72 |
| None vs. B-B | 100 | 56.97 | 43.03 | 12.76 | 3 | 3 | 3.373 | 72 |
| None vs. B - M | 100 | 56.97 | 43.03 | 12.76 | 3 | 3 | 3.373 | 72 |
| None vs. M - - | 100 | 56.73 | 43.27 | 12.76 | 3 | 3 | 3.392 | 72 |
| None vs. M - M | 100 | 56.73 | 43.27 | 12.76 | 3 | 3 | 3.392 | 72 |
| None vs. M - B | 100 | 56.73 | 43.27 | 12.76 | 3 | 3 | 3.392 | 72 |
| None vs. BM - - | 100 | 23.37 | 76.63 | 12.76 | 3 | 3 | 6.007 | 72 |
| None vs. BM - BM | 100 | 23.37 | 76.63 | 12.76 | 3 | 3 | 6.007 | 72 |
| | | | | | | | | |
| Day7 | | | | | | | | |
| None vs. B - - | 100 | 59.56 | 40.44 | 12.76 | 3 | 3 | 3.17 | 72 |
| None vs. B-B | 100 | 43.04 | 56.96 | 12.76 | 3 | 3 | 4.465 | 72 |
| None vs. B - M | 100 | 38.54 | 61.46 | 12.76 | 3 | 3 | 4.818 | 72 |
| None vs. M - - | 100 | 70.64 | 29.36 | 12.76 | 3 | 3 | 2.302 | 72 |
| None vs. M - M | 100 | 42.01 | 57.99 | 12.76 | 3 | 3 | 4.546 | 72 |
| None vs. M - B | 100 | 58.18 | 41.82 | 12.76 | 3 | 3 | 3.278 | 72 |
| None vs. BM - - | 100 | 26.37 | 73.63 | 12.76 | 3 | 3 | 5.772 | 72 |
| None vs. BM - BM | 100 | 10.11 | 89.89 | 12.76 | 3 | 3 | 7.046 | 72 |
| | | | | | | | | |
| Day11 | | | | | | | | |
| None vs. B - - | 100 | 91.16 | 8.837 | 12.76 | 3 | 3 | 0.6928 | 72 |
| None vs. B-B | 100 | 53.72 | 46.28 | 12.76 | 3 | 3 | 3.628 | 72 |
| None vs. B - M | 100 | 69.3 | 30.7 | 12.76 | 3 | 3 | 2.407 | 72 |
| None vs. M - - | 100 | 93.05 | 6.949 | 12.76 | 3 | 3 | 0.5448 | 72 |
| None vs. M - M | 100 | 73.08 | 26.92 | 12.76 | 3 | 3 | 2.11 | 72 |
| None vs. M - B | 100 | 62.06 | 37.94 | 12.76 | 3 | 3 | 2.974 | 72 |
| None vs. BM - - | 100 | 52.34 | 47.66 | 12.76 | 3 | 3 | 3.736 | 72 |
| None vs. BM - BM | 100 | 5.561 | 94.44 | 12.76 | 3 | 3 | 7.403 | 72 |
| | | | | | | | | |
| Day14 | | | | | | | | |
| None vs. B - - | 100 | 94.1 | 5.896 | 12.76 | 3 | 3 | 0.4622 | 72 |
| None vs. B-B | 100 | 65.75 | 34.25 | 12.76 | 3 | 3 | 2.685 | 72 |
| None vs. B - M | 100 | 80.4 | 19.6 | 12.76 | 3 | 3 | 1.537 | 72 |
| None vs. M - - | 100 | 76.55 | 23.45 | 12.76 | 3 | 3 | 1.838 | 72 |
| None vs. M - M | 100 | 67.67 | 32.33 | 12.76 | 3 | 3 | 2.535 | 72 |
| None vs. M - B | 100 | 60.73 | 39.27 | 12.76 | 3 | 3 | 3.078 | 72 |
| None vs. BM - - | 100 | 71.47 | 28.53 | 12.76 | 3 | 3 | 2.236 | 72 |
| None vs. BM - BM | 100 | 6.758 | 93.24 | 12.76 | 3 | 3 | 7.309 | 72 |

## Slide 3
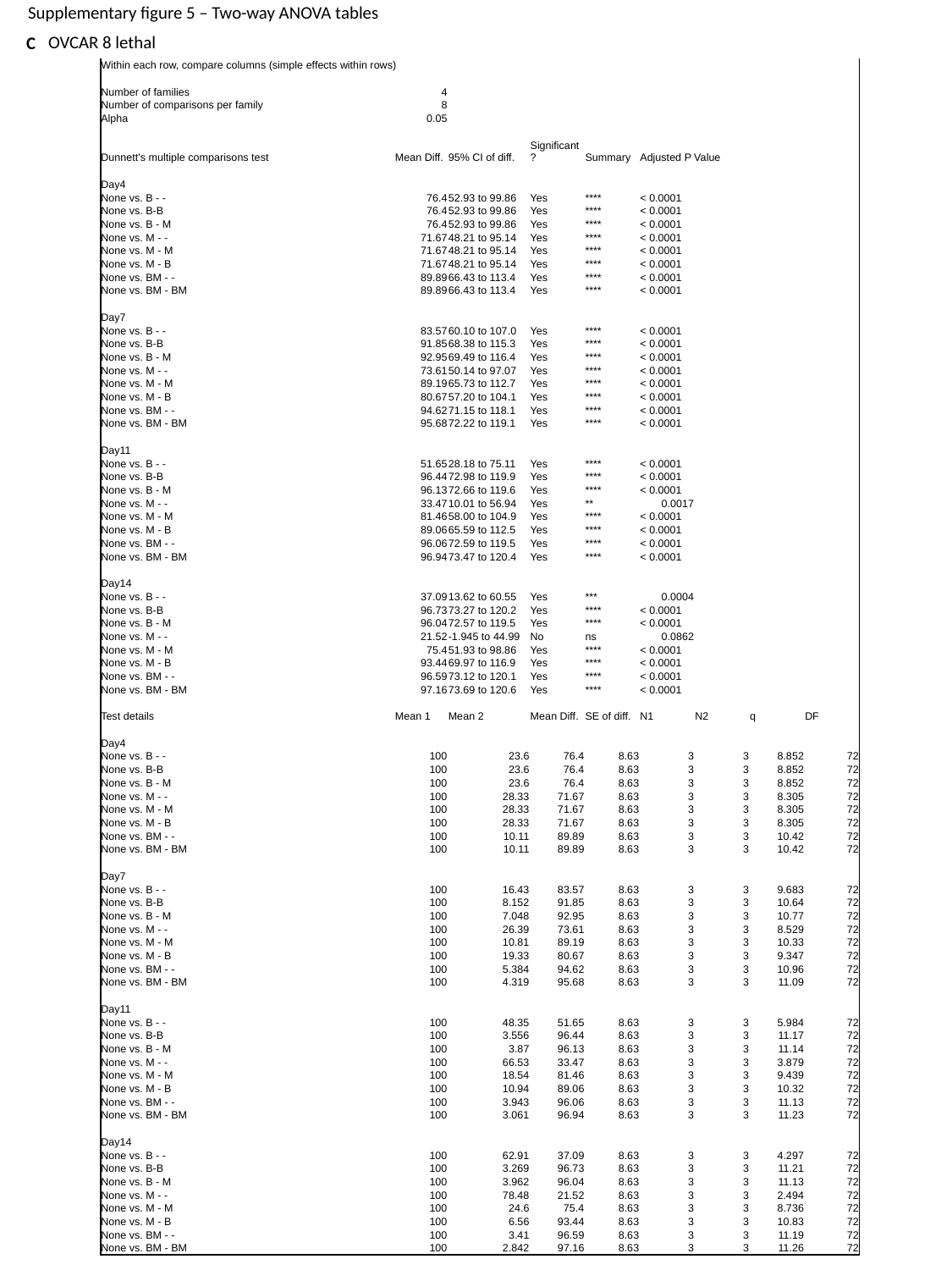

Supplementary figure 5 – Two-way ANOVA tables
OVCAR 8 lethal
C
| Within each row, compare columns (simple effects within rows) | | | | | | | | |
| --- | --- | --- | --- | --- | --- | --- | --- | --- |
| | | | | | | | | |
| Number of families | 4 | | | | | | | |
| Number of comparisons per family | 8 | | | | | | | |
| Alpha | 0.05 | | | | | | | |
| | | | | | | | | |
| Dunnett's multiple comparisons test | Mean Diff. | 95% CI of diff. | Significant? | Summary | Adjusted P Value | | | |
| | | | | | | | | |
| Day4 | | | | | | | | |
| None vs. B - - | 76.4 | 52.93 to 99.86 | Yes | \*\*\*\* | < 0.0001 | | | |
| None vs. B-B | 76.4 | 52.93 to 99.86 | Yes | \*\*\*\* | < 0.0001 | | | |
| None vs. B - M | 76.4 | 52.93 to 99.86 | Yes | \*\*\*\* | < 0.0001 | | | |
| None vs. M - - | 71.67 | 48.21 to 95.14 | Yes | \*\*\*\* | < 0.0001 | | | |
| None vs. M - M | 71.67 | 48.21 to 95.14 | Yes | \*\*\*\* | < 0.0001 | | | |
| None vs. M - B | 71.67 | 48.21 to 95.14 | Yes | \*\*\*\* | < 0.0001 | | | |
| None vs. BM - - | 89.89 | 66.43 to 113.4 | Yes | \*\*\*\* | < 0.0001 | | | |
| None vs. BM - BM | 89.89 | 66.43 to 113.4 | Yes | \*\*\*\* | < 0.0001 | | | |
| | | | | | | | | |
| Day7 | | | | | | | | |
| None vs. B - - | 83.57 | 60.10 to 107.0 | Yes | \*\*\*\* | < 0.0001 | | | |
| None vs. B-B | 91.85 | 68.38 to 115.3 | Yes | \*\*\*\* | < 0.0001 | | | |
| None vs. B - M | 92.95 | 69.49 to 116.4 | Yes | \*\*\*\* | < 0.0001 | | | |
| None vs. M - - | 73.61 | 50.14 to 97.07 | Yes | \*\*\*\* | < 0.0001 | | | |
| None vs. M - M | 89.19 | 65.73 to 112.7 | Yes | \*\*\*\* | < 0.0001 | | | |
| None vs. M - B | 80.67 | 57.20 to 104.1 | Yes | \*\*\*\* | < 0.0001 | | | |
| None vs. BM - - | 94.62 | 71.15 to 118.1 | Yes | \*\*\*\* | < 0.0001 | | | |
| None vs. BM - BM | 95.68 | 72.22 to 119.1 | Yes | \*\*\*\* | < 0.0001 | | | |
| | | | | | | | | |
| Day11 | | | | | | | | |
| None vs. B - - | 51.65 | 28.18 to 75.11 | Yes | \*\*\*\* | < 0.0001 | | | |
| None vs. B-B | 96.44 | 72.98 to 119.9 | Yes | \*\*\*\* | < 0.0001 | | | |
| None vs. B - M | 96.13 | 72.66 to 119.6 | Yes | \*\*\*\* | < 0.0001 | | | |
| None vs. M - - | 33.47 | 10.01 to 56.94 | Yes | \*\* | 0.0017 | | | |
| None vs. M - M | 81.46 | 58.00 to 104.9 | Yes | \*\*\*\* | < 0.0001 | | | |
| None vs. M - B | 89.06 | 65.59 to 112.5 | Yes | \*\*\*\* | < 0.0001 | | | |
| None vs. BM - - | 96.06 | 72.59 to 119.5 | Yes | \*\*\*\* | < 0.0001 | | | |
| None vs. BM - BM | 96.94 | 73.47 to 120.4 | Yes | \*\*\*\* | < 0.0001 | | | |
| | | | | | | | | |
| Day14 | | | | | | | | |
| None vs. B - - | 37.09 | 13.62 to 60.55 | Yes | \*\*\* | 0.0004 | | | |
| None vs. B-B | 96.73 | 73.27 to 120.2 | Yes | \*\*\*\* | < 0.0001 | | | |
| None vs. B - M | 96.04 | 72.57 to 119.5 | Yes | \*\*\*\* | < 0.0001 | | | |
| None vs. M - - | 21.52 | -1.945 to 44.99 | No | ns | 0.0862 | | | |
| None vs. M - M | 75.4 | 51.93 to 98.86 | Yes | \*\*\*\* | < 0.0001 | | | |
| None vs. M - B | 93.44 | 69.97 to 116.9 | Yes | \*\*\*\* | < 0.0001 | | | |
| None vs. BM - - | 96.59 | 73.12 to 120.1 | Yes | \*\*\*\* | < 0.0001 | | | |
| None vs. BM - BM | 97.16 | 73.69 to 120.6 | Yes | \*\*\*\* | < 0.0001 | | | |
| | | | | | | | | |
| Test details | Mean 1 | Mean 2 | Mean Diff. | SE of diff. | N1 | N2 | q | DF |
| | | | | | | | | |
| Day4 | | | | | | | | |
| None vs. B - - | 100 | 23.6 | 76.4 | 8.63 | 3 | 3 | 8.852 | 72 |
| None vs. B-B | 100 | 23.6 | 76.4 | 8.63 | 3 | 3 | 8.852 | 72 |
| None vs. B - M | 100 | 23.6 | 76.4 | 8.63 | 3 | 3 | 8.852 | 72 |
| None vs. M - - | 100 | 28.33 | 71.67 | 8.63 | 3 | 3 | 8.305 | 72 |
| None vs. M - M | 100 | 28.33 | 71.67 | 8.63 | 3 | 3 | 8.305 | 72 |
| None vs. M - B | 100 | 28.33 | 71.67 | 8.63 | 3 | 3 | 8.305 | 72 |
| None vs. BM - - | 100 | 10.11 | 89.89 | 8.63 | 3 | 3 | 10.42 | 72 |
| None vs. BM - BM | 100 | 10.11 | 89.89 | 8.63 | 3 | 3 | 10.42 | 72 |
| | | | | | | | | |
| Day7 | | | | | | | | |
| None vs. B - - | 100 | 16.43 | 83.57 | 8.63 | 3 | 3 | 9.683 | 72 |
| None vs. B-B | 100 | 8.152 | 91.85 | 8.63 | 3 | 3 | 10.64 | 72 |
| None vs. B - M | 100 | 7.048 | 92.95 | 8.63 | 3 | 3 | 10.77 | 72 |
| None vs. M - - | 100 | 26.39 | 73.61 | 8.63 | 3 | 3 | 8.529 | 72 |
| None vs. M - M | 100 | 10.81 | 89.19 | 8.63 | 3 | 3 | 10.33 | 72 |
| None vs. M - B | 100 | 19.33 | 80.67 | 8.63 | 3 | 3 | 9.347 | 72 |
| None vs. BM - - | 100 | 5.384 | 94.62 | 8.63 | 3 | 3 | 10.96 | 72 |
| None vs. BM - BM | 100 | 4.319 | 95.68 | 8.63 | 3 | 3 | 11.09 | 72 |
| | | | | | | | | |
| Day11 | | | | | | | | |
| None vs. B - - | 100 | 48.35 | 51.65 | 8.63 | 3 | 3 | 5.984 | 72 |
| None vs. B-B | 100 | 3.556 | 96.44 | 8.63 | 3 | 3 | 11.17 | 72 |
| None vs. B - M | 100 | 3.87 | 96.13 | 8.63 | 3 | 3 | 11.14 | 72 |
| None vs. M - - | 100 | 66.53 | 33.47 | 8.63 | 3 | 3 | 3.879 | 72 |
| None vs. M - M | 100 | 18.54 | 81.46 | 8.63 | 3 | 3 | 9.439 | 72 |
| None vs. M - B | 100 | 10.94 | 89.06 | 8.63 | 3 | 3 | 10.32 | 72 |
| None vs. BM - - | 100 | 3.943 | 96.06 | 8.63 | 3 | 3 | 11.13 | 72 |
| None vs. BM - BM | 100 | 3.061 | 96.94 | 8.63 | 3 | 3 | 11.23 | 72 |
| | | | | | | | | |
| Day14 | | | | | | | | |
| None vs. B - - | 100 | 62.91 | 37.09 | 8.63 | 3 | 3 | 4.297 | 72 |
| None vs. B-B | 100 | 3.269 | 96.73 | 8.63 | 3 | 3 | 11.21 | 72 |
| None vs. B - M | 100 | 3.962 | 96.04 | 8.63 | 3 | 3 | 11.13 | 72 |
| None vs. M - - | 100 | 78.48 | 21.52 | 8.63 | 3 | 3 | 2.494 | 72 |
| None vs. M - M | 100 | 24.6 | 75.4 | 8.63 | 3 | 3 | 8.736 | 72 |
| None vs. M - B | 100 | 6.56 | 93.44 | 8.63 | 3 | 3 | 10.83 | 72 |
| None vs. BM - - | 100 | 3.41 | 96.59 | 8.63 | 3 | 3 | 11.19 | 72 |
| None vs. BM - BM | 100 | 2.842 | 97.16 | 8.63 | 3 | 3 | 11.26 | 72 |

## Slide 4
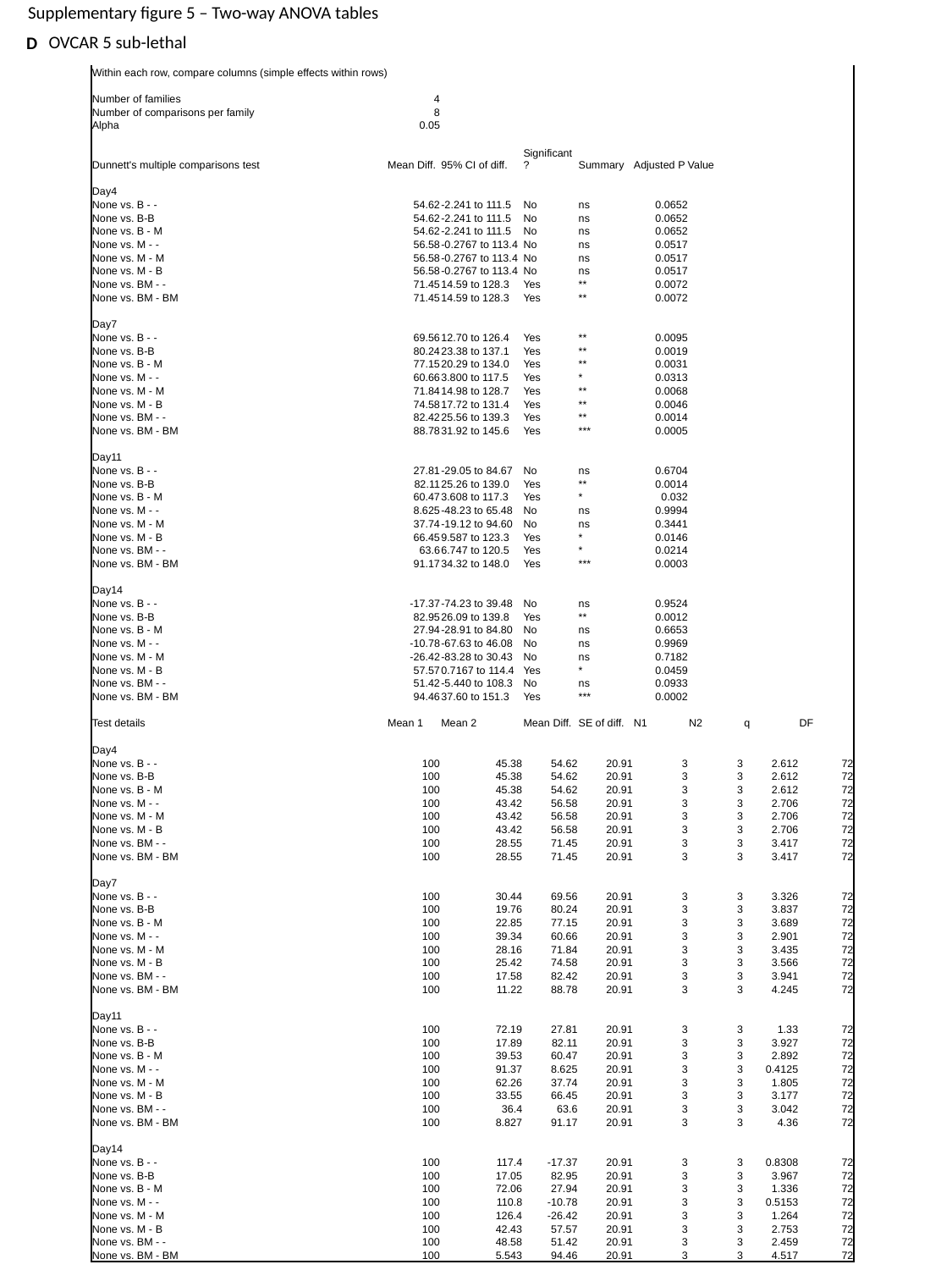

Supplementary figure 5 – Two-way ANOVA tables
OVCAR 5 sub-lethal
D
| Within each row, compare columns (simple effects within rows) | | | | | | | | |
| --- | --- | --- | --- | --- | --- | --- | --- | --- |
| | | | | | | | | |
| Number of families | 4 | | | | | | | |
| Number of comparisons per family | 8 | | | | | | | |
| Alpha | 0.05 | | | | | | | |
| | | | | | | | | |
| Dunnett's multiple comparisons test | Mean Diff. | 95% CI of diff. | Significant? | Summary | Adjusted P Value | | | |
| | | | | | | | | |
| Day4 | | | | | | | | |
| None vs. B - - | 54.62 | -2.241 to 111.5 | No | ns | 0.0652 | | | |
| None vs. B-B | 54.62 | -2.241 to 111.5 | No | ns | 0.0652 | | | |
| None vs. B - M | 54.62 | -2.241 to 111.5 | No | ns | 0.0652 | | | |
| None vs. M - - | 56.58 | -0.2767 to 113.4 | No | ns | 0.0517 | | | |
| None vs. M - M | 56.58 | -0.2767 to 113.4 | No | ns | 0.0517 | | | |
| None vs. M - B | 56.58 | -0.2767 to 113.4 | No | ns | 0.0517 | | | |
| None vs. BM - - | 71.45 | 14.59 to 128.3 | Yes | \*\* | 0.0072 | | | |
| None vs. BM - BM | 71.45 | 14.59 to 128.3 | Yes | \*\* | 0.0072 | | | |
| | | | | | | | | |
| Day7 | | | | | | | | |
| None vs. B - - | 69.56 | 12.70 to 126.4 | Yes | \*\* | 0.0095 | | | |
| None vs. B-B | 80.24 | 23.38 to 137.1 | Yes | \*\* | 0.0019 | | | |
| None vs. B - M | 77.15 | 20.29 to 134.0 | Yes | \*\* | 0.0031 | | | |
| None vs. M - - | 60.66 | 3.800 to 117.5 | Yes | \* | 0.0313 | | | |
| None vs. M - M | 71.84 | 14.98 to 128.7 | Yes | \*\* | 0.0068 | | | |
| None vs. M - B | 74.58 | 17.72 to 131.4 | Yes | \*\* | 0.0046 | | | |
| None vs. BM - - | 82.42 | 25.56 to 139.3 | Yes | \*\* | 0.0014 | | | |
| None vs. BM - BM | 88.78 | 31.92 to 145.6 | Yes | \*\*\* | 0.0005 | | | |
| | | | | | | | | |
| Day11 | | | | | | | | |
| None vs. B - - | 27.81 | -29.05 to 84.67 | No | ns | 0.6704 | | | |
| None vs. B-B | 82.11 | 25.26 to 139.0 | Yes | \*\* | 0.0014 | | | |
| None vs. B - M | 60.47 | 3.608 to 117.3 | Yes | \* | 0.032 | | | |
| None vs. M - - | 8.625 | -48.23 to 65.48 | No | ns | 0.9994 | | | |
| None vs. M - M | 37.74 | -19.12 to 94.60 | No | ns | 0.3441 | | | |
| None vs. M - B | 66.45 | 9.587 to 123.3 | Yes | \* | 0.0146 | | | |
| None vs. BM - - | 63.6 | 6.747 to 120.5 | Yes | \* | 0.0214 | | | |
| None vs. BM - BM | 91.17 | 34.32 to 148.0 | Yes | \*\*\* | 0.0003 | | | |
| | | | | | | | | |
| Day14 | | | | | | | | |
| None vs. B - - | -17.37 | -74.23 to 39.48 | No | ns | 0.9524 | | | |
| None vs. B-B | 82.95 | 26.09 to 139.8 | Yes | \*\* | 0.0012 | | | |
| None vs. B - M | 27.94 | -28.91 to 84.80 | No | ns | 0.6653 | | | |
| None vs. M - - | -10.78 | -67.63 to 46.08 | No | ns | 0.9969 | | | |
| None vs. M - M | -26.42 | -83.28 to 30.43 | No | ns | 0.7182 | | | |
| None vs. M - B | 57.57 | 0.7167 to 114.4 | Yes | \* | 0.0459 | | | |
| None vs. BM - - | 51.42 | -5.440 to 108.3 | No | ns | 0.0933 | | | |
| None vs. BM - BM | 94.46 | 37.60 to 151.3 | Yes | \*\*\* | 0.0002 | | | |
| | | | | | | | | |
| Test details | Mean 1 | Mean 2 | Mean Diff. | SE of diff. | N1 | N2 | q | DF |
| | | | | | | | | |
| Day4 | | | | | | | | |
| None vs. B - - | 100 | 45.38 | 54.62 | 20.91 | 3 | 3 | 2.612 | 72 |
| None vs. B-B | 100 | 45.38 | 54.62 | 20.91 | 3 | 3 | 2.612 | 72 |
| None vs. B - M | 100 | 45.38 | 54.62 | 20.91 | 3 | 3 | 2.612 | 72 |
| None vs. M - - | 100 | 43.42 | 56.58 | 20.91 | 3 | 3 | 2.706 | 72 |
| None vs. M - M | 100 | 43.42 | 56.58 | 20.91 | 3 | 3 | 2.706 | 72 |
| None vs. M - B | 100 | 43.42 | 56.58 | 20.91 | 3 | 3 | 2.706 | 72 |
| None vs. BM - - | 100 | 28.55 | 71.45 | 20.91 | 3 | 3 | 3.417 | 72 |
| None vs. BM - BM | 100 | 28.55 | 71.45 | 20.91 | 3 | 3 | 3.417 | 72 |
| | | | | | | | | |
| Day7 | | | | | | | | |
| None vs. B - - | 100 | 30.44 | 69.56 | 20.91 | 3 | 3 | 3.326 | 72 |
| None vs. B-B | 100 | 19.76 | 80.24 | 20.91 | 3 | 3 | 3.837 | 72 |
| None vs. B - M | 100 | 22.85 | 77.15 | 20.91 | 3 | 3 | 3.689 | 72 |
| None vs. M - - | 100 | 39.34 | 60.66 | 20.91 | 3 | 3 | 2.901 | 72 |
| None vs. M - M | 100 | 28.16 | 71.84 | 20.91 | 3 | 3 | 3.435 | 72 |
| None vs. M - B | 100 | 25.42 | 74.58 | 20.91 | 3 | 3 | 3.566 | 72 |
| None vs. BM - - | 100 | 17.58 | 82.42 | 20.91 | 3 | 3 | 3.941 | 72 |
| None vs. BM - BM | 100 | 11.22 | 88.78 | 20.91 | 3 | 3 | 4.245 | 72 |
| | | | | | | | | |
| Day11 | | | | | | | | |
| None vs. B - - | 100 | 72.19 | 27.81 | 20.91 | 3 | 3 | 1.33 | 72 |
| None vs. B-B | 100 | 17.89 | 82.11 | 20.91 | 3 | 3 | 3.927 | 72 |
| None vs. B - M | 100 | 39.53 | 60.47 | 20.91 | 3 | 3 | 2.892 | 72 |
| None vs. M - - | 100 | 91.37 | 8.625 | 20.91 | 3 | 3 | 0.4125 | 72 |
| None vs. M - M | 100 | 62.26 | 37.74 | 20.91 | 3 | 3 | 1.805 | 72 |
| None vs. M - B | 100 | 33.55 | 66.45 | 20.91 | 3 | 3 | 3.177 | 72 |
| None vs. BM - - | 100 | 36.4 | 63.6 | 20.91 | 3 | 3 | 3.042 | 72 |
| None vs. BM - BM | 100 | 8.827 | 91.17 | 20.91 | 3 | 3 | 4.36 | 72 |
| | | | | | | | | |
| Day14 | | | | | | | | |
| None vs. B - - | 100 | 117.4 | -17.37 | 20.91 | 3 | 3 | 0.8308 | 72 |
| None vs. B-B | 100 | 17.05 | 82.95 | 20.91 | 3 | 3 | 3.967 | 72 |
| None vs. B - M | 100 | 72.06 | 27.94 | 20.91 | 3 | 3 | 1.336 | 72 |
| None vs. M - - | 100 | 110.8 | -10.78 | 20.91 | 3 | 3 | 0.5153 | 72 |
| None vs. M - M | 100 | 126.4 | -26.42 | 20.91 | 3 | 3 | 1.264 | 72 |
| None vs. M - B | 100 | 42.43 | 57.57 | 20.91 | 3 | 3 | 2.753 | 72 |
| None vs. BM - - | 100 | 48.58 | 51.42 | 20.91 | 3 | 3 | 2.459 | 72 |
| None vs. BM - BM | 100 | 5.543 | 94.46 | 20.91 | 3 | 3 | 4.517 | 72 |

## Slide 5
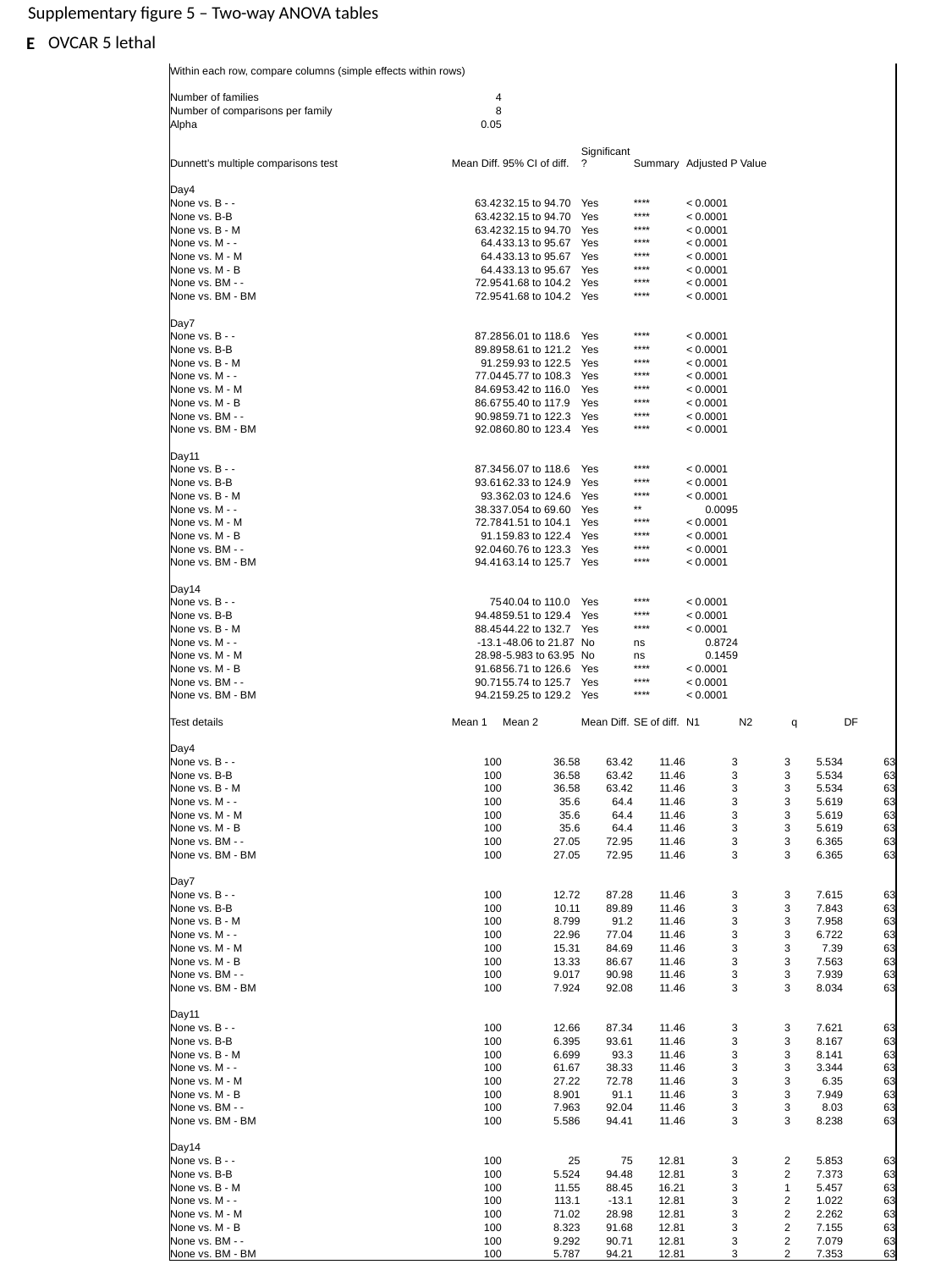

Supplementary figure 5 – Two-way ANOVA tables
OVCAR 5 lethal
E
| Within each row, compare columns (simple effects within rows) | | | | | | | | |
| --- | --- | --- | --- | --- | --- | --- | --- | --- |
| | | | | | | | | |
| Number of families | 4 | | | | | | | |
| Number of comparisons per family | 8 | | | | | | | |
| Alpha | 0.05 | | | | | | | |
| | | | | | | | | |
| Dunnett's multiple comparisons test | Mean Diff. | 95% CI of diff. | Significant? | Summary | Adjusted P Value | | | |
| | | | | | | | | |
| Day4 | | | | | | | | |
| None vs. B - - | 63.42 | 32.15 to 94.70 | Yes | \*\*\*\* | < 0.0001 | | | |
| None vs. B-B | 63.42 | 32.15 to 94.70 | Yes | \*\*\*\* | < 0.0001 | | | |
| None vs. B - M | 63.42 | 32.15 to 94.70 | Yes | \*\*\*\* | < 0.0001 | | | |
| None vs. M - - | 64.4 | 33.13 to 95.67 | Yes | \*\*\*\* | < 0.0001 | | | |
| None vs. M - M | 64.4 | 33.13 to 95.67 | Yes | \*\*\*\* | < 0.0001 | | | |
| None vs. M - B | 64.4 | 33.13 to 95.67 | Yes | \*\*\*\* | < 0.0001 | | | |
| None vs. BM - - | 72.95 | 41.68 to 104.2 | Yes | \*\*\*\* | < 0.0001 | | | |
| None vs. BM - BM | 72.95 | 41.68 to 104.2 | Yes | \*\*\*\* | < 0.0001 | | | |
| | | | | | | | | |
| Day7 | | | | | | | | |
| None vs. B - - | 87.28 | 56.01 to 118.6 | Yes | \*\*\*\* | < 0.0001 | | | |
| None vs. B-B | 89.89 | 58.61 to 121.2 | Yes | \*\*\*\* | < 0.0001 | | | |
| None vs. B - M | 91.2 | 59.93 to 122.5 | Yes | \*\*\*\* | < 0.0001 | | | |
| None vs. M - - | 77.04 | 45.77 to 108.3 | Yes | \*\*\*\* | < 0.0001 | | | |
| None vs. M - M | 84.69 | 53.42 to 116.0 | Yes | \*\*\*\* | < 0.0001 | | | |
| None vs. M - B | 86.67 | 55.40 to 117.9 | Yes | \*\*\*\* | < 0.0001 | | | |
| None vs. BM - - | 90.98 | 59.71 to 122.3 | Yes | \*\*\*\* | < 0.0001 | | | |
| None vs. BM - BM | 92.08 | 60.80 to 123.4 | Yes | \*\*\*\* | < 0.0001 | | | |
| | | | | | | | | |
| Day11 | | | | | | | | |
| None vs. B - - | 87.34 | 56.07 to 118.6 | Yes | \*\*\*\* | < 0.0001 | | | |
| None vs. B-B | 93.61 | 62.33 to 124.9 | Yes | \*\*\*\* | < 0.0001 | | | |
| None vs. B - M | 93.3 | 62.03 to 124.6 | Yes | \*\*\*\* | < 0.0001 | | | |
| None vs. M - - | 38.33 | 7.054 to 69.60 | Yes | \*\* | 0.0095 | | | |
| None vs. M - M | 72.78 | 41.51 to 104.1 | Yes | \*\*\*\* | < 0.0001 | | | |
| None vs. M - B | 91.1 | 59.83 to 122.4 | Yes | \*\*\*\* | < 0.0001 | | | |
| None vs. BM - - | 92.04 | 60.76 to 123.3 | Yes | \*\*\*\* | < 0.0001 | | | |
| None vs. BM - BM | 94.41 | 63.14 to 125.7 | Yes | \*\*\*\* | < 0.0001 | | | |
| | | | | | | | | |
| Day14 | | | | | | | | |
| None vs. B - - | 75 | 40.04 to 110.0 | Yes | \*\*\*\* | < 0.0001 | | | |
| None vs. B-B | 94.48 | 59.51 to 129.4 | Yes | \*\*\*\* | < 0.0001 | | | |
| None vs. B - M | 88.45 | 44.22 to 132.7 | Yes | \*\*\*\* | < 0.0001 | | | |
| None vs. M - - | -13.1 | -48.06 to 21.87 | No | ns | 0.8724 | | | |
| None vs. M - M | 28.98 | -5.983 to 63.95 | No | ns | 0.1459 | | | |
| None vs. M - B | 91.68 | 56.71 to 126.6 | Yes | \*\*\*\* | < 0.0001 | | | |
| None vs. BM - - | 90.71 | 55.74 to 125.7 | Yes | \*\*\*\* | < 0.0001 | | | |
| None vs. BM - BM | 94.21 | 59.25 to 129.2 | Yes | \*\*\*\* | < 0.0001 | | | |
| | | | | | | | | |
| Test details | Mean 1 | Mean 2 | Mean Diff. | SE of diff. | N1 | N2 | q | DF |
| | | | | | | | | |
| Day4 | | | | | | | | |
| None vs. B - - | 100 | 36.58 | 63.42 | 11.46 | 3 | 3 | 5.534 | 63 |
| None vs. B-B | 100 | 36.58 | 63.42 | 11.46 | 3 | 3 | 5.534 | 63 |
| None vs. B - M | 100 | 36.58 | 63.42 | 11.46 | 3 | 3 | 5.534 | 63 |
| None vs. M - - | 100 | 35.6 | 64.4 | 11.46 | 3 | 3 | 5.619 | 63 |
| None vs. M - M | 100 | 35.6 | 64.4 | 11.46 | 3 | 3 | 5.619 | 63 |
| None vs. M - B | 100 | 35.6 | 64.4 | 11.46 | 3 | 3 | 5.619 | 63 |
| None vs. BM - - | 100 | 27.05 | 72.95 | 11.46 | 3 | 3 | 6.365 | 63 |
| None vs. BM - BM | 100 | 27.05 | 72.95 | 11.46 | 3 | 3 | 6.365 | 63 |
| | | | | | | | | |
| Day7 | | | | | | | | |
| None vs. B - - | 100 | 12.72 | 87.28 | 11.46 | 3 | 3 | 7.615 | 63 |
| None vs. B-B | 100 | 10.11 | 89.89 | 11.46 | 3 | 3 | 7.843 | 63 |
| None vs. B - M | 100 | 8.799 | 91.2 | 11.46 | 3 | 3 | 7.958 | 63 |
| None vs. M - - | 100 | 22.96 | 77.04 | 11.46 | 3 | 3 | 6.722 | 63 |
| None vs. M - M | 100 | 15.31 | 84.69 | 11.46 | 3 | 3 | 7.39 | 63 |
| None vs. M - B | 100 | 13.33 | 86.67 | 11.46 | 3 | 3 | 7.563 | 63 |
| None vs. BM - - | 100 | 9.017 | 90.98 | 11.46 | 3 | 3 | 7.939 | 63 |
| None vs. BM - BM | 100 | 7.924 | 92.08 | 11.46 | 3 | 3 | 8.034 | 63 |
| | | | | | | | | |
| Day11 | | | | | | | | |
| None vs. B - - | 100 | 12.66 | 87.34 | 11.46 | 3 | 3 | 7.621 | 63 |
| None vs. B-B | 100 | 6.395 | 93.61 | 11.46 | 3 | 3 | 8.167 | 63 |
| None vs. B - M | 100 | 6.699 | 93.3 | 11.46 | 3 | 3 | 8.141 | 63 |
| None vs. M - - | 100 | 61.67 | 38.33 | 11.46 | 3 | 3 | 3.344 | 63 |
| None vs. M - M | 100 | 27.22 | 72.78 | 11.46 | 3 | 3 | 6.35 | 63 |
| None vs. M - B | 100 | 8.901 | 91.1 | 11.46 | 3 | 3 | 7.949 | 63 |
| None vs. BM - - | 100 | 7.963 | 92.04 | 11.46 | 3 | 3 | 8.03 | 63 |
| None vs. BM - BM | 100 | 5.586 | 94.41 | 11.46 | 3 | 3 | 8.238 | 63 |
| | | | | | | | | |
| Day14 | | | | | | | | |
| None vs. B - - | 100 | 25 | 75 | 12.81 | 3 | 2 | 5.853 | 63 |
| None vs. B-B | 100 | 5.524 | 94.48 | 12.81 | 3 | 2 | 7.373 | 63 |
| None vs. B - M | 100 | 11.55 | 88.45 | 16.21 | 3 | 1 | 5.457 | 63 |
| None vs. M - - | 100 | 113.1 | -13.1 | 12.81 | 3 | 2 | 1.022 | 63 |
| None vs. M - M | 100 | 71.02 | 28.98 | 12.81 | 3 | 2 | 2.262 | 63 |
| None vs. M - B | 100 | 8.323 | 91.68 | 12.81 | 3 | 2 | 7.155 | 63 |
| None vs. BM - - | 100 | 9.292 | 90.71 | 12.81 | 3 | 2 | 7.079 | 63 |
| None vs. BM - BM | 100 | 5.787 | 94.21 | 12.81 | 3 | 2 | 7.353 | 63 |
