# Supplementary material for: Identification of therapeutic targets applicable to clinical strategies in ovarian cancer
Source: BMC Cancer. 2016 Aug 24;16(1):678. doi: 10.1186/s12885-016-2675-5 (PMC4997769; doi:10.1186/s12885-016-2675-5)
Supplement: Additional file 12: Table S2. — Candidate shRNAs identified in A2780. (DOCX 40 kb) [file 12885_2016_2675_MOESM12_ESM.docx]

**Additional file 12 Table S2A:** Statistical analysis for cellular viability: p-values for cellular viability across all cell lines compared against siNeg normalized to test value=1.

siRNA #1:


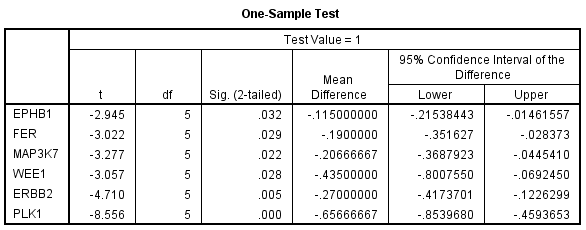


siRNA #2:


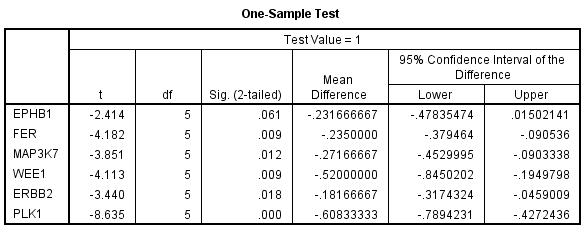


**Additional file 12 Table S2B:** Statistical analysis for cellular viability: p-values for cellular viability in each cell line and siRNA with a combined mean from #1 and #2 compared against siNeg normalized to test value=1.

|  | **Ovcar3** | **Ovcar8** | **Igrov1** | **Ovcar5** | **Skov3** | **A2780** |
| --- | --- | --- | --- | --- | --- | --- |
| **siEPBH1** | .435 | .246 | .044 | .344 | .100 | .164 |
| **siERBB2** | .083 | .126 | .063 | .295 | .274 | .033 |
| **siFER** | .016 | .159 | .019 | .136 | .368 | .136 |
| **siMAP3K7** | .042 | .228 | .056 | .111 | .312 | .037 |
| **siPLK1** | .039 | .011 | .025 | .000 | .045 | .022 |
| **siWEE1** | .025 | .032 | .010 | .131 | .356 | .152 |
